# Temperature‐Resilient Reconfigurable Physical Unclonable Function Driven by Pulse Modulation Using CMOS‐Integrated Spintronic Chips

**DOI:** 10.1002/advs.74926

**Published:** 2026-03-27

**Authors:** Min Wang, Chuanpeng Jiang, Zhaohao Wang, Zhengyi Hou, Zhongkui Zhang, Yuanfu Zhao, Hong‐xi Liu, Weisheng Zhao

**Affiliations:** ^1^ School of Integrated Circuit Science and Engineering Beihang University Beijing China; ^2^ Hangzhou International Innovation Institute Beihang University Hangzhou China; ^3^ Beijing Microelectronics Technology Institute Beijing China; ^4^ Truth Memory Corporation Beijing China

**Keywords:** CMOS‐integrated chip, hardware security, reconfigurable physical unclonable function, SOT‐MRAM, thermal resilience

## Abstract

Hardware‐based security primitives have become critical to enhancing information security in the Internet of Things (IoT) era. Physical unclonable functions (PUFs) utilize the inherent variations in the manufacturing process to generate cryptographic keys unique to a device. Reconfigurable PUFs can update cryptographic keys for enhanced security in dynamic operational scenarios involving huge amounts of data, which makes them suitable for implementation in CMOS‐integrated spin‐orbit torque magnetic random access memory (SOT‐MRAM) chips. However, a key challenge is achieving real‐time reconfiguration independent of the environmental conditions, particularly the operating temperature. We propose a dual‐pulse reconfiguration strategy for PUF design in CMOS‐integrated SOT‐MRAM chips that effectively widens the operating window and achieves resilience across a wide range of operating temperatures without the need for dynamic feedback that overly complicates circuit design. The proposed strategy lays a solid foundation for the next generation of hardware‐based security primitives to protect IoT architectures.

## Graphical abstract

A reconfigurable physical unclonable function is developed using CMOS‐integrated SOT‐MRAM chips, leveraging a dual‐pulse strategy and offering enhanced environmental robustness. A temperature‐compensation effect arising from the CMOS transistor and SOT‐MTJ is revealed and established as a key prerequisite for thermal resilience. This design achieves a universal reconfiguration window from ‐40 to 125, enabling a feasible hardware security primitive toward IoT deployment.

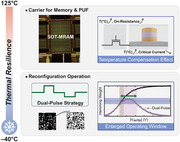

## 1 Introduction

Information security requires multilayered encryption spanning both software and hardware. The expansion of the Internet of Things (IoT) has made millions of edge devices and systems vulnerable to cyber threats, making information security at the hardware level increasingly critical [1, 2, 3]. Key hardware‐based security primitives include the Trusted Platform Module, Hardware Security Module, and Physical Unclonable Function (PUF). In particular, PUFs are a lightweight and cost‐effective solution that generate cryptographic keys specific to a chip based on inherent variations in the manufacturing process [4, 5, 6, 7]. These cryptographic keys are in the form of challenge–response pairs (CRPs), which serve as a digital fingerprint and guarantee the uniqueness and non‐clonability of the chip.

PUF designs based on complementary metal–oxide–semiconductor (CMOS) technology feature circuit compatibility and employ inherent variations in the manufacturing process as a static entropy source [8, 9, 10, 11, 12, 13]. To satisfy the requirements of high‐frequency interaction scenarios, stronger PUF designs are being investigated that expand the CRP space exponentially. However, once the cryptographic key is compromised, fixed CRPs are at risk of being permanently invalidated [14]. The above limitations have given rise to the reconfigurable PUF, which is an emerging security primitive that utilizes dynamic entropy sources to update the CRP space. This not only expands the CRP space but also enhances security and flexibility against machine learning attacks and untrusted manufacturing risk [15, 16, 17]. Nonetheless, CMOS‐based PUFs hold weak cycle‐to‐cycle (C2C) variability and cannot support higher‐order reconfiguration counts.

Advances in non‐volatile memory (NVM) have introduced novel entropy sources that allow reconfigurable PUFs to be implemented with enhanced security metrics [18, 19, 20, 21, 22, 23, 24] in various memory technologies, which include the ferroelectric field‐effect transistor (FeFET) [25, 26, 27, 28], phase‐change random access memory (PC‐RAM) [29], resistance random access memory (RRAM) [16, 30, 31, 32], spin‐transfer torque magnetic random access memory (STT‐MRAM) [33, 34, 35, 36, 37], and spin‐orbit torque magnetic random access memory (SOT‐MRAM) [17, 38, 39, 40, 41, 42]. For NVM‐based PUFs, the balance between endurance and reliability is an important consideration. SOT‐MRAM is considered an ideal replacement for static random access memory because of its high reliability, high speed, and low power consumption [43, 44]. SOT‐MRAM stores binary data in a magnetic tunnel junction (MTJ), which provides inherent dynamic entropy for excellent C2C variability and a physical basis for key regeneration [45, 46] as well as unlimited endurance for high‐order reconfiguration counts [47]. Thus, SOT‐MRAM is an ideal carrier for multifunctional PUFs, which are considered a prime candidate for the next generation of hardware‐based security primitives.

Previous studies on reconfigurable PUFs for SOT‐MRAM chips have generally adopted C2C variability with stochastic characteristics based on perpendicular and in‐plane anisotropic MTJs, such as variations induced by a switching current ($I_{c}$) [17], domain wall nonlinear dynamics [48], chiral domain wall motion [38], and random switching enhanced by self‐write‐back (SWB) [49]. Beyond stochastic SOT switching dynamics, recent studies have employed complex magnetic textures as entropy sources. For instance, He et al. demonstrated a novel approach based on spontaneously formed labyrinth domain patterns [24]. Unfortunately, these designs remain at the device or small‐scale array level, and practical applications will inevitably require integrated control circuits such as CMOS modules. There is still a lack of analysis on the chip‐level reconfiguration and external environment robustness of CMOS‐integrated SOT‐MRAM chips, for which temperature variations can cause shifts in both the transistor and MTJ parameters.

Two main metrics are affected by the temperature: the read reliability and real‐time reconfiguration. Various studies on PUFs have focused on enhancing the read reliability over a wide temperature range, such as by SWB [10, 49], temporal majority voting (TMV) [11, 50, 51], unsteady bit masking or discard [11, 12, 50, 51], and feedback compensation [13, 51, 52, 53]. Meanwhile, fewer studies have considered ensuring that real‐time reconfiguration can be performed over a wide temperature range. Real‐time reconfiguration requires a stable operating window, and a major challenge is the temperature‐dependent drift of the critical current for the MTJ. Moreover, conventional feedback compensation techniques are not applicable to CMOS‐integrated SOT‐MRAM chips, so new schemes for reconfigurable PUFs need to be designed. However, the physical mechanism by which the temperature affects CMOS‐integrated SOT‐MRAM chips is still unclear, and the real‐time reconfiguration against external temperature fluctuations faces two major bottlenecks: (i) a narrow operating window that requires highly precise circuit design; (ii) the shift in dynamic probability with the operating temperature that increases the complexity of the compensation circuit.

In this work, we propose and experimentally demonstrate a temperature‐resilient reconfigurable PUF design in CMOS‐integrated SOT‐MRAM chips. The novel dual‐pulse reconfiguration strategy, as a fully‐electrical manipulation, increases the operating window for on‐demand reconfiguration and compensates for shifts in the operating temperature. This work further reveals the temperature compensation effect, a unique chip‐level coupling phenomenon that cannot be observed at the device level. By combining a dual‐pulse reconfiguration strategy with the temperature compensation effect, this work systematically addresses the thermal resilience bottleneck and establishes a universal operating window spanning –40 to 125 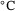, suitable for industrial‐grade hardware security. The reconfigurable PUF design is a feasible and streamlined solution for enhanced robustness against temperature with high potential for lightweight hardware‐based security primitives.

## 2 Results

**FIGURE 1:**
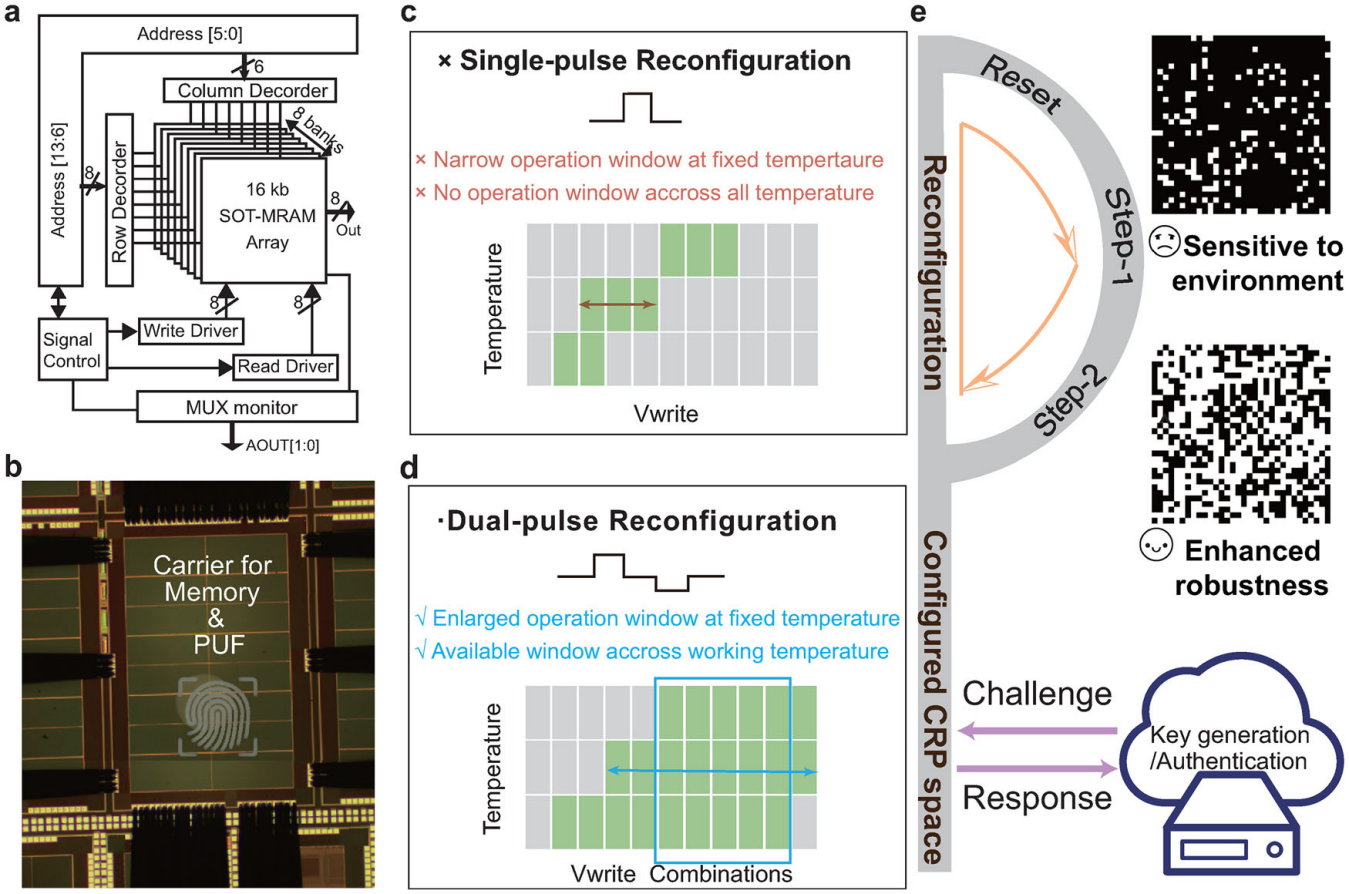
Overview of the proposed reconfigurable PUF. (a) Architecture of a 128 kb SOT‐MRAM chip comprising eight banks. (b) Optical microscopy image of the SOT‐MRAM chip during the chip‐probing test. Overview of (c) the conventional single‐pulse reconfiguration strategy and (d) the proposed dual‐pulse reconfiguration strategy. (e) Workflow of the proposed reconfigurable PUF and its authentication protocol. The inserted binary bitmaps indicate the generated PUF responses after the first and second set pulses.

Figure 1a illustrates the architecture of a 128 kb SOT‐MRAM chip that was fabricated by using 180 nm CMOS technology on an 8‐inch platform [54] and integrates eight banks of 16 kb SOT arrays, a read circuit, a write driver, decoders, and buffers. Notably, the multiplexer module is designed to allow access to bit cells in analog mode. In other words, bit cells can flexibly operate with either digital or analog signals depending on whether the multiplexer module is disabled or enabled. One bit of data is stored and read out from a pair of MTJs; in memory mode or after SWB operation, the data are stored in complementary pairs. This pair design contributes to differential read circuits and high read reliability, while also preventing side‐channel attacks because of the weak fluctuations in the current when 0 or 1 is read. This type of MTJ has in‐plane magnetic anisotropy, which has been demonstrated to be a reliable carrier because of its all‐electric write operation and high back‐end‐of‐line compatibility.

The SOT‐MRAM chip can serve as a shared carrier for both memory and PUF modes. The functionality of the chip was verified by generating a shmoo plot of the write success rate (WSR) under various writing conditions (Figure S1), and we have verified memory mode in previous studies [54, 55]. Regarding PUF mode, the SOT‐MRAM chip features both static entropy and dynamic entropy that are induced by the fabrication process and thermal noise, respectively. Some of the metrics used to evaluate memory mode can also be implemented in PUF mode. For example, retention of over 10 years indicates long‐term reliability in PUF mode. The endurance was found to be more than $1\times 10^{18}$ [56], which ensures high‐order reconfiguration cycles. To further characterize device‐to‐device variation, a statistical analysis of the MTJ resistance is presented in Figure S3. Near‐Gaussian resistance distributions were observed, and comparisons of the same‐state resistances within the unit resulted in a near‐50% ‘0/1’ distribution. Static entropy sources are sufficient for PUF implementation; to further expand the CRP space, dynamic entropy sources are more suitable.

In this study, experiments were conducted by using the chip‐probing test, as shown in Figure 1b. The write pulse width was set to 20 ns by default. The analog voltage generated by the chip‐probing test machine was applied through the multiplexer module as the write voltage ($V_{write}$). Figure 1c shows the conventional single‐pulse reconfiguration strategy for PUFs, which involves reset and single‐step set pulses. The green regions represent the valid operation window where the WSR meets the requirement for PUF reconfiguration. Affected by the thermal disturbance field, the WSR is a sigmoid‐like and monotonically increasing function of the write voltage, which inherently limits the width of the operating window at a fixed temperature. As the temperature changes, the operation window drifts and results in no crossover between different temperatures. Thus, the single‐pulse strategy lacks a universal operating window for on‐demand reconfiguration across operating temperatures.

Because the SOT‐MRAM chip may undergo reconfiguration at any moment, it is not possible to wait for the temperature to return to room temperature. Therefore, the single‐pulse reconfiguration strategy is difficult to integrate with conventional temperature feedback techniques. Figure 1d shows the proposed dual‐pulse reconfiguration strategy for widening the operating window and enhancing robustness against temperature. This strategy employs two pulses of opposite polarities and decreasing amplitudes, which modifies the monotonically increasing probability curve of the conventional single‐pulse reconfiguration strategy. The green area is notably wider horizontally, which indicates a broader operating window at a fixed temperature. The blue box indicates a universal working window across the entire temperature range from –40 to 125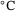. Thus, the dual‐pulse reconfiguration strategy effectively leverages probability curve regulation to generate a stable reconfiguration window.

Figure 1e illustrates the workflow of the reconfigurable PUF in an SOT‐MRAM chip. First, the chip is initialized to 1 values (i.e., FF polarized writing). Next, the first set pulse of 00 polarity is applied to form a map of stochastic states, which may be affected by the operating temperature and deviate from the ideal distribution. Finally, a second set pulse of FF polarity is applied to correct the data distribution. The above process is denoted as FF–00–FF polarized writing, while the reverse is denoted as 00–FF–00 polarized writing. The related authentication protocol is as follows. When the response needs to be refreshed, the reconfiguration starts, and the response stored by the SOT‐MRAM chip is written randomly via the dual‐pulse reconfiguration strategy. After reconfiguration, the SOT‐MRAM chip stores stable responses and interacts with external devices for authentication in the form of a CRP. Chips from the same manufacturer or after a new reconfiguration can vary greatly in CRP space, which greatly reduces the risk of being copied or predicted and ensures system security. To distinguish between single‐bit and chip‐level statistics, we employed three complementary metrics: the Hamming weight (HW) to quantify the distribution of 1 values at the chip level, the WSR compared to the initialization state, and the switching probability $P_{sw}$ to characterize single‐bit probabilistic behavior. In the single‐pulse reconfiguration strategy, the HW directly corresponds to the WSR during 00–FF processes. For FF–00 processes, HW = 1–WSR. The bit error rate (BER) was used as a metric to evaluate the read reliability.

### 2.1 Experimental Validation at Room Temperature

**FIGURE 2:**
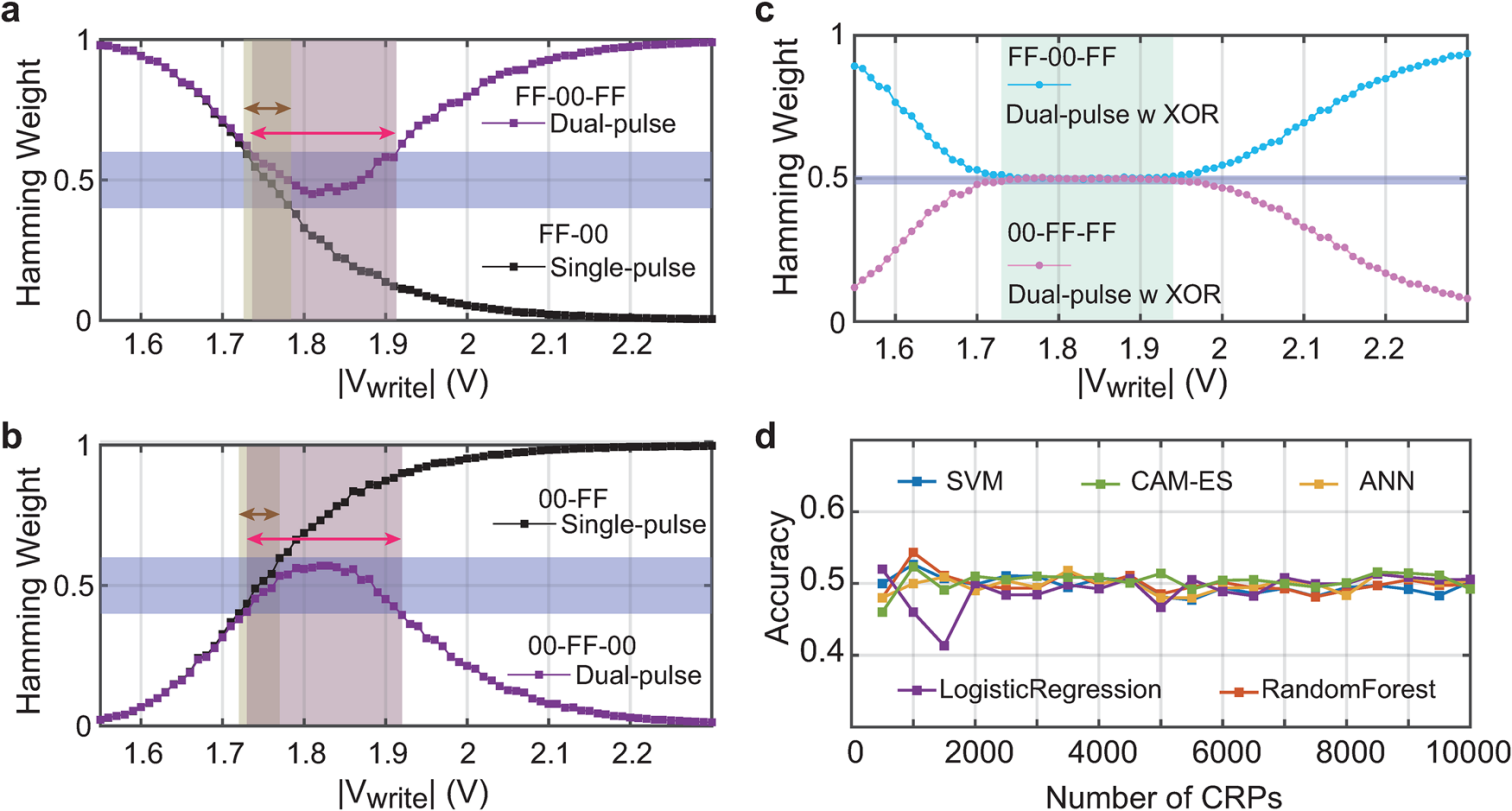
PUF performance with the dual‐pulse reconfiguration strategy. Hamming weight (HW) as a function of the write voltage with different reconfiguration strategies: (a) FF–00 (single‐pulse) and FF–00–FF (dual‐pulse) polarized writing and (b) 00–FF (single‐pulse) and 00–FF–00 (dual‐pulse) polarized writing. The target operating window is indicated in blue. β = 0.15 V was used. (c) Enhancement of the HW with XOR postprocessing. (d) Robustness against machine learning attacks targeting the predicted PUF response mapping. The prediction accuracy of five machine learning models was ∼50%.

We experimentally validated the proposed reconfigurable PUF by first evaluating the operating window at room temperature. Figure 2a shows the measured HW according to the reconfiguration strategy with FF–00 (single‐pulse) and FF–00–FF(dual‐pulse) polarized writing. With the single‐pulse reconfiguration strategy, the HW increased monotonically with the write voltage, similar to a sigmoid‐like function. With the dual‐pulse reconfiguration strategy, the HW approximated a parabolic function because of the correction by the second set pulse. The first and second set pulses held different polarities, and the relationship between the amplitudes of the first set (V1) and second set (V2) can be defined as

(1) |V2|=|V1|−β

where β is a correction coefficient. As shown in Figure 2b, the same behavior was observed for 00–FF and 00–FF–00 polarized writing. The preliminary target operating window (blue color) limited the HW to 0.4–0.6, which is a prerequisite for XOR postprocessing (see Section S3). The XOR postprocessing obfuscates PUF data by addressing spatial correlations from process deviations and enhancing key metrics. Figure 2c shows the ideal HW and large operating window obtained with XOR postprocessing. Thus, the proposed dual‐pulse reconfiguration strategy facilitated a wider operating window while the generated PUF responses retained excellent unpredictability. To evaluate security, five classical algorithms were modeled and executed (details in Section S4) [34, 57, 58, 59, 60, 61, 62]. As both the differential digital readout and SOT‐based write paths exhibit negligible data‐correlated side‐channel leakage, learning the challenge–response mapping constitutes the primary viable attack surface. The raw prediction accuracy was 55.29% without postprocessing (Figure S7), which confirmed the entropy from C2C and device‐to‐device (D2D) variability. As shown in Figure 2d, a nearly ideal random guessing level (50.03%) was achieved after XOR postprocessing, which confirmed the robustness of the reconfigurable PUF against machine learning attacks.

In terms of uniformity, the dual‐pulse reconfiguration strategy combined with XOR postprocessing led to a near‐ideal Gauss distribution of reconfigurable PUF responses with μ=0.5001 and σ= 0.042, where the fitted distribution was tightly clustered around μ (Figures S8 and S9). The randomness and unpredictability of the reconfigurable PUF responses were further demonstrated by the results of the Automatic Correction Function test (Figure S10) and NIST SP800‐22 test (Table S1). The enhanced reliability of our design was also explored in terms of the TMV and SWB operations. TMV is an error correction technique that performs multiple read operations in a selected address and uses most of the readout data as the final data. TMV is suitable for energy‐saving scenarios because the reading process consumes less energy than the writing process. SWB rewrites the memory cell deterministically according to the readout data, which mitigates unstable bits and optimizes reliability metrics such as the intra‐Hamming distance (intra‐HD) and BER. The inter‐/intra‐HD ratio was approximately infinite at 1500 (Figure S11a,b), and the average BER was $3.29\times 10^{-5}$. In addition, the inter‐die HD (μ = 0.5009, σ = 0.0437) demonstrated the excellent uniqueness of the proposed reconfigurable PUF (Figure S11b).

### 2.2 Demonstration of Reconfigurability

**FIGURE 3:**
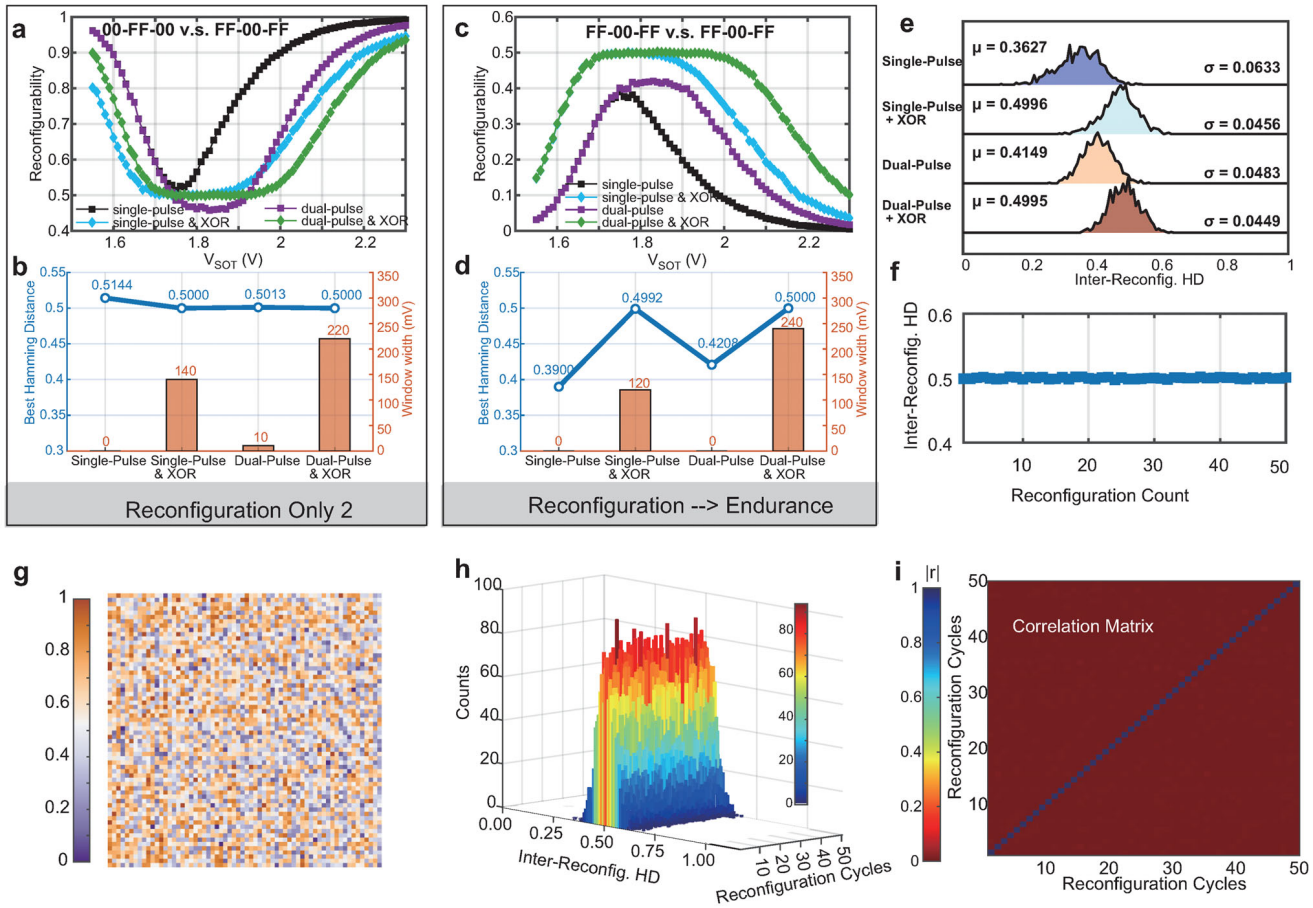
Reconfigurability with the proposed dual‐pulse reconfiguration strategy. (a,b) Reconfigurability between 00–FF–00 and FF–00–FF polarized writing. (c,d) Reconfigurability with FF–00–FF writing repeated. (e) Inter‐reconfiguration HD using the single‐ and dual‐pulse reconfiguration strategies without/with XOR postprocessing. The write voltage was set to 1.8 V for the single‐pulse reconfiguration as well as the first voltage for the dual‐pulse reconfiguration. β was set to 0.15 V, and the second voltage was set to 1.65 V. (f) Mean value of the inter‐reconfiguration HD for 50 reconfigurations. (g) Extracted switching probability map; only 4 kb is shown here. (h) Distribution of the inter‐reconfiguration HD for 50 reconfigurations. (i) Correlation matrix of reconfigured keys. The results in sub‐figures (f)‐(i) are for FF–00–FF polarized writing.

The reconfigurability is defined by the inter‐reconfiguration HD, which reflects the uniqueness among reconfigured keys. One way to realize reconfigurability is to use the C2C and D2D variability without severe spatial domain dependence. As shown in Figure 3a,b, 00–FF and FF–00 polarized writing with the single‐pulse reconfiguration strategy can achieve two reconfigurations with a near‐ideal inter‐reconfiguration HD. The dual‐pulse reconfiguration strategy can increase the size of the operating window but suffers from the inherent limit that it allows only two reconfigurations, i.e., configured CRP by 00–FF–00 (CRP@00–FF–00) and FF–00–FF (CRP@FF–00–FF) writing. As shown in Figure 3c,d, the inter‐reconfiguration HD between the first‐time configuration (CRP@1st‐00–FF–00) and the second‐time configuration (CRP@2nd‐00–FF–00) was 0.4, which is insufficient for the construction of two separate keys. To solve this issue, XOR postprocessing was applied to improve the inter‐reconfiguration HD (green lines in Figure 3c).

The mutual HDs among CRP@1st‐00–FF–00, @2nd‐00–FF–00, until @N‐th‐00–FF–00 were sufficient, which indicated that these N sets of CRPs can be considered as independent PUFs. Thus, the proposed dual‐pulse reconfiguration strategy achieved nearly unlimited cycling by leveraging the unlimited endurance of SOT‐MRAM [56]. Theoretically, $2^{128}$ combinations can be generated in an unpredictable form, and the strong endurance of SOT‐MRAM is sufficient to support real‐time reconfiguration for the entire life cycle (Figures S12 and S13 and Section S6). Figure 3e shows that the reconfigurability with the proposed strategy combining dual‐pulse reconfiguration and XOR postprocessing holds a good mean and narrow distribution. Thus, the proposed strategy achieves a large operating window width, ideal inter‐reconfiguration HD, and unlimited reconfiguration.

Figure 3f shows that the mean value of the inter‐reconfiguration HD among 50 reconfigured keys was calculated as 0.5000 within a range of 0.4973−0.5022, which demonstrates the stability and effectiveness of the proposed strategy. Notably, the strategy supports bidirectional reconfiguration (i.e., multiple writing of FF–00–FF and/or 00–FF–00). The reconfigurability originates from the relatively independent distributions of C2C and D2D variability in the temporal and spatial domains, reinforced by XOR postprocessing, as shown by the switching probability map in Figure 3g. Figure 3h presents the distribution of the inter‐reconfiguration HD for 50 reconfigured keys. The correlation matrix in Figure 3i confirms the nonlinearity and noncorrelation among these reconfigured keys. Generating keys using 00–FF–00 polarized writing also resulted in good reconfigurability and independence (Figures S14 and S15). We demonstrate the feasibility and reconfigurability of the proposed reconfigurable PUF design. The 128 kb capacity of the SOT‐MRAM chip enabled a large CRP space and unlimited reconfiguration. The uniqueness, randomness, and reliability of the reconfigurable PUF were verified at the chip level.

### 2.3 Resilience Against Temperature

**FIGURE 4:**
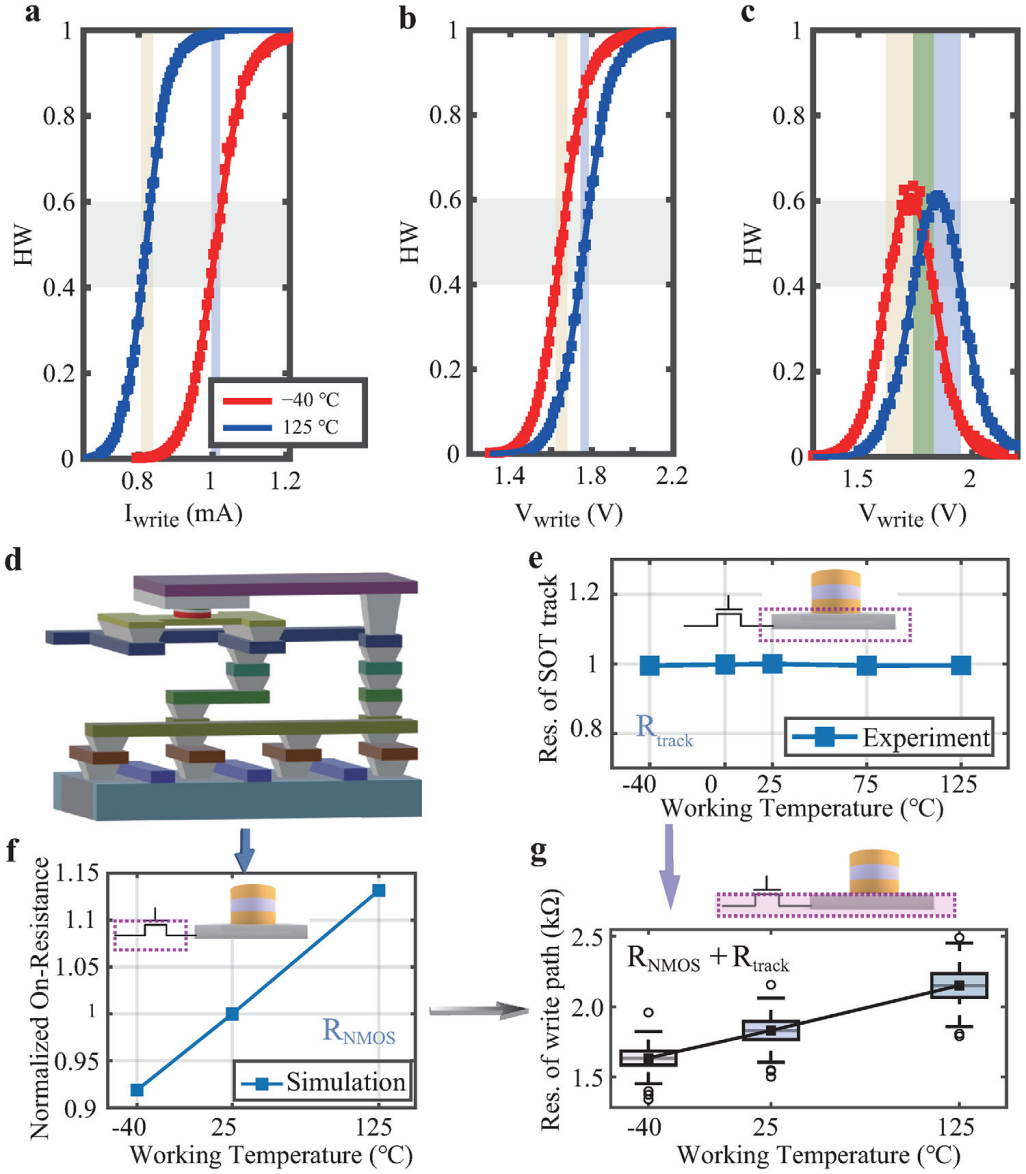
Temperature compensation effect in the reconfigurable PUF for an SOT‐MRAM chip. Probability curves as functions of the (a) write current and (b) write voltage with the single‐pulse reconfiguration strategy and (c) write voltage with the dual‐pulse reconfiguration strategy. Experiments were performed at –40 and 125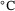. The write current was calculated from the write voltage and the average write‐path resistance. (d) 3D layout of the two‐transistor one‐MTJ (2T1J) structure of the SOT‐MRAM chip. (e) SOT track resistance according to the temperature of single MTJs. (f) On‐resistance for the write transistor according to temperature. (g) Measured write‐path resistance.

Next, the impacts of temperature on the reconfiguration and performance of the proposed reconfigurable PUF were evaluated. As shown in Figure 4a, the critical switching current is inherently sensitive to the operating temperature and tends to decrease with an increase in temperature. This phenomenon can be attributed to the reduced thermal energy barrier at high temperatures, which facilitates magnetization reversal. As shown in Figure 4b, the single‐pulse reconfiguration strategy resulted in smaller deviation in the voltage‐dependent curves at –40 and 125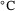, which indicates a smaller difference in the critical write voltage across operating temperatures. However, overlapping operating windows could not be achieved, and uniform pulse settings could not be achieved across the temperature range, even with XOR postprocessing. As shown in Figure 4c, the dual‐pulse reconfiguration strategy also resulted in a smaller deviation, as well as a common operating window across various temperatures. The second pulse corrected the deviation in the first pulse. Optimizing the combination of the first and second pulses resulted in an operating window that spanned the entire temperature range (green color). Thus, the temperature resilience for the reconfigurability of the proposed reconfigurable PUF was verified at the chip level.

To our knowledge, the temperature compensation effect in CMOS‐integrated SOT‐MRAM chips has not been explored. Here, we first discuss the prerequisites for the dual‐pulse reconfiguration strategy to be feasible and then explain the temperature compensation effect. Based on the range of applicability of XOR postprocessing, the voltage ranges of the target window can be denoted as $\left[a_{1},a_{2}\right]$ at –40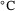 and as $\left[b_{1},b_{2}\right]$ at 125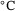. It is a prerequisite that the intersection of these two ranges exists:

(2) $$\max\left(a_{1},b_{1}\right)\leq \min\left(a_{2},b_{2}\right)$$

In other words, the intersection of the two voltage ranges ($\left[a_{1},a_{2}\right]\cap \left[b_{1},b_{2}\right]$) must not be empty.

For the SOT‐MRAM chip, a two‐transistor‐one‐MTJ (2T1J) structure was adopted, where each MTJ was associated with a write transistor and read transistor, as shown in Figure 4d [63]. The write‐path resistance mainly comprises the on‐resistance of the write transistor and the resistance of the SOT track. Figure 4e,f show the relationships of these two resistances with the temperature. The SOT track resistance was measured for single MTJs fabricated on the same platform. The SOT track resistance of annealed β−phase tungsten (β−W) exhibited a weak thermal dependence (Table S6), which is consistent with the previous report [64]. The transistors exhibited a positive temperature coefficient with the on‐resistance increasing with temperature (see Figure 4f). This behavior can be attributed to the reduction in carrier mobility with increasing temperature, which decreased the charge carrier density per unit area in a fixed electric field and thus increased the on‐resistance. If the on‐resistance and SOT track resistance are combined, then the results indicate that the total write‐path resistance increases with temperature. As shown in Figure 4g, measurements in analog mode confirmed this positive temperature dependence. Therefore, while the critical write current decreases with temperature, the corresponding deviation in the write voltage is compensated for by an increase in the on‐resistance, which stabilizes the voltage‐dependent characteristics.

The proposed reconfigurable PUF addresses both of the main issues of the environmental temperature: real‐time reconfiguration and read reliability. The SOT‐MRAM chip demonstrated excellent reconfigurability from –40 to 125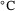 (Figure S16), which proved that the dual‐pulse reconfiguration strategy can be used regardless of the operating temperature while increasing the operating window. Thus, the two major bottlenecks concerning real‐time reconfiguration are resolved. In terms of read reliability, the SOT‐MRAM chip demonstrated improved BER reliability at non‐nominal temperatures and power supply VDD conditions (Figure S17), which was enhanced by SWB operation and the differential read circuit compared to the baseline without SWB (Figure S18).

### 2.4 Numerical Modeling

We developed a numerical model to optimize the pulse combinations for the dual‐pulse reconfiguration strategy (β = |V1| – |V2|). β determines the width of the operating window, which can be defined as the intersection of the WSR and the target window. The final WSR can be expressed as a composite of two WSR functions for the single pulses WSR1(V) and WSR2(V). Because the switching probabilities of two write operations are statistically independent (see Section S12), the final WSR of the dual‐pulse reconfiguration strategy (F(V1,β)) can be expressed as

(3) F(V1,β)=WSR1(V1)−WSR1(V1)·WSR2(V2)

**FIGURE 5:**
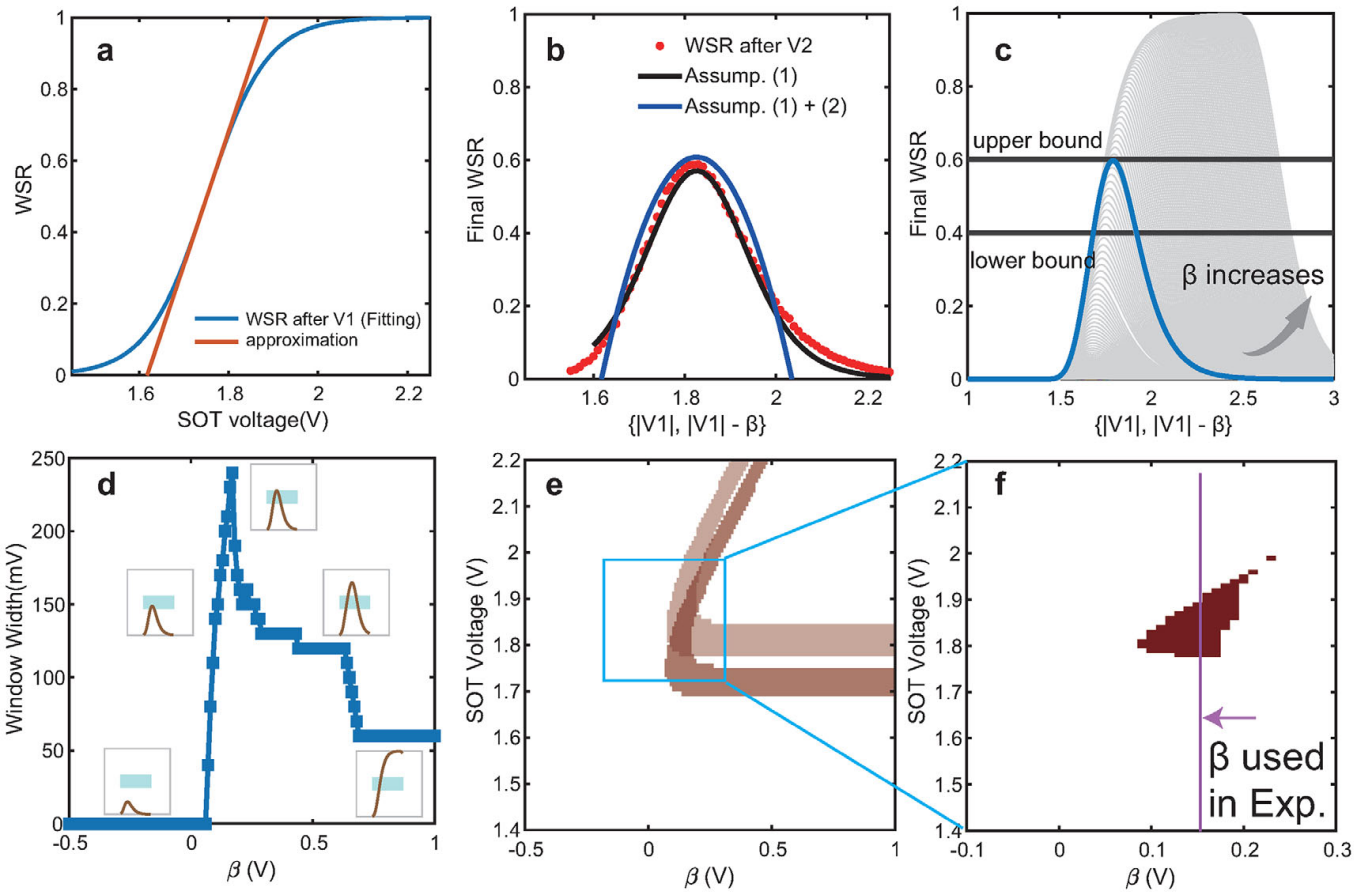
Numerical modeling of the dual‐pulse reconfiguration strategy and solution for β. (a) Simplified model for WSR curves with the single‐pulse reconfiguration strategy. (b) Experimental and predicted final WSR with the dual‐pulse reconfiguration strategy. (c) Final WSR with the dual‐pulse reconfiguration strategy using different values of β based on WSR1 at room temperature. (d) Width of the operating window as a function of β. Insets show five typical cases. (e) Phase diagrams of available pulse combinations as functions of β and the V1 in the cases of –40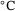 and 125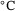. The overlapped region represents the common operating window across operating temperatures. (f) Magnified view of the common operating window and location of β used in experiments (0.15 V).

Figure 5a shows the WSR function of single‐pulse writing, which has a sigmoid‐like shape and behaves similarly with both 00–FF and FF–00 polarized writing. This results in Assumption 1: Single pulses of different polarities correspond to the same WSR function (i.e., WSR1(V)≈WSR2(V)). Thus, Equation (3) can be revised as

(4) F(V1,β)=WSR1(V1)−WSR1(V1)·WSR1(V1−β)

The corresponding result in Figure 5b (black line) shows the close agreement between the numerical model and experimental values, which indicates that Assumption 1 is reasonable.

The calculation can be further simplified by Assumption 2: Within a specific range, the voltage is linearly related to the WSR with a slope of k, which is represented by the tangent line as shown in Figure 5a. k mainly depends on the process variation (see Section S11). Combining Assumptions 1 and 2 results in

(5) F=WSR1(V1)−WSR1(V1)·(WSR1(V1)−k·β)

where WSR1 reduces to a unitary function. As shown in Figure 5b, the corresponding result (blue line) simplifies to a univariate quadratic curve. In this case, the value of β can be determined by solving for the coordinates of the extremes, which results in the optimal value for β being 0.147 V (Section S12). Considering the accuracy of the source meter, β was set to a value of 0.15 V in the experiment.

Figure 5c presents a parameter sweep of β for the dual‐pulse reconfiguration strategy, with the model of Equation (4). Figure 5d summarizes the widths of the operating window with different β and the write voltage limited to < 2.4 V. When β was below a certain threshold value, no operating window existed. When β exceeded the threshold value, the operating window initially increased until it reached a peak width. Further increasing β caused the operating window to start decreasing in width. As shown by the inset, the extremes of the F(V1,β) curve gradually approached the lower bound of the target window and then the upper bound before overflowing the target window.

Based on the temperature range, phase diagrams of available pulse combinations as a function of β versus V1 were generated for –40 and 125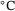 and overlapped, as shown in Figure 5e. Colored pixels indicate where F(V1,β) lies within the target window, while the overlapping region represents the common operating window across temperatures. Figure 5f shows a magnified view of the common operating window. Both the calculated value (0.147 V) and experimental value (0.15 V) of β are within this region and close to the central area, demonstrating strong agreement between the experimental data and simulation results.

Additionally, ten chips from the same batch were tested. The results demonstrated that all chips could be reconstructed across the entire temperature range. This confirmed the consistency of the β value and applicability of the dual‐pulse reconfiguration strategy (Table S7 and Section S11). This cross‐chip consistency allows global deployment of the reconfigurable PUF within the same batch, which eliminates the need for customizing the design of each chip. Multichip experimental results further validated the presence of the temperature compensation effect in CMOS‐integrated SOT‐MRAM chips, which is an intrinsic physical characteristic of the integrated platform rather than an isolated phenomenon. With the prospect of fully functional chips (Figure S22), the dual‐pulse reconfiguration strategy can be implemented by the bandgap reference and reference buffer circuits, which are both industry‐standard circuit modules.

## 3 Discussion

We developed a reconfigurable PUF for CMOS‐integrated SOT‐MRAM chips that can operate over a wide temperature range and reconfigure in real‐time, which is a pivotal advance for hardware‐based security primitives. The proposed dual‐pulse reconfiguration strategy modifies the monotonically increasing relationship between the switching probability and electrical excitation of the conventional single‐pulse strategy to increase the size of the operating window. The CMOS‐integrated SOT‐MRAM chip offers a unique temperature compensation effect that facilitates real‐time reconfiguration from –40 to 125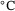. With increasing temperature, the increased write‐path resistance counteracts the decreased critical write current of the MTJ, resulting in a smaller offset of the critical write voltage compared to that of the critical write current. The proposed design demonstrated notable improvements in various performance metrics as summarized in Table 1, which highlights its potential application to improving the reliability of hardware‐based security. The C2C and D2D variability was comprehensively investigated, and the results showed that combining the dual‐pulse reconfiguration strategy with XOR postprocessing resulted in robustness against machine learning attacks and excellent reconfigurability. The read reliability is enhanced by a differential read circuit and SWB, which results in an approximately infinite inter/intra‐HD ratio and ultralow BER.

**TABLE 1:** Comparison with the state‐of‐the‐art reconfigurable PUF designs.

	This Work	TCAS‐I'22 [42]	Adv. Mat.'25 [24]	ESSERC'25 [37]	ISSCC'19 [30]	VLSI'24 [32]	Nat. Comm.'25 [26]
Tech. Node	180 nm	40 nm (Simu.)	Device	40 nm	130 nm	28 nm	28 nm
Carrier	Y‐type SOT‐MRAM	Z‐type SOT‐MRAM	Labyrinth domains	STT‐MRAM	RRAM	RRAM	FeFET
Entropy Source	Stochastic Switching	Thermal Noise	Thermal‐driven Domain Dynamics	Stochastic Switching	Post‐process Randomness	SwitchingCompetition	Cycle‐to‐cycle Variation
Inter‐Reconfig. HD	0.4995	0.5028	0.4993	0.456	0.4729	—	0.5002
Temp. Range (Read) ( 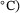	−40∼125	−25∼100	−40∼125	−25∼125	25∼150	−40∼125	25∼85
Reconfigurability Across Temp. Range	Yes	—	Yes	—	—	—	—
BER	$3.29\times 10^{-5}$	$<10^{-5}$	0.058	$2.5\times 10^{-5}$	$<6.1\times 10^{-6}$	$5\times 10^{-9}$	0
Uniformity	0.5001	0.4992	∼0.5	—	0.5001	—	0.5000
Inter‐Die HD	0.5009	0.5004	—	0.5026	0.4999	0.5001	0.4998
Entropy	0.9886^a)^	0.9937^a)^	—	—	—	0.994^a)^	∼1
Energy Consumption (pJ/bit)	0.24^b)^, ^d)^; 2.59^c)^, ^d)^	—	$3.5\times 10^{-4}$ ^c)^	0.178 ^b)^	3.028	0.023 ^b)^	0.016 ^b)^
Anti‐ML Attack	Yes	—	Yes	—	—	Yes	Yes

a) Minimum entropy;

b) Read energy;

c) Reconfiguration energy or reset energy;

d) Energy extrapolated from technology‐node scaling [65] and SOT‐MTJ dimension miniaturization (Figure S21).

Our work distinguishes itself from state‐of‐the‐art reconfigurable PUFs by leveraging a unique physical temperature compensation mechanism together with a novel, fully electrical dual‐pulse reconfiguration strategy. The proposed design demonstrates the thermal resilience of reconfiguration as well as the temperature reliability of read operations, enabling robust in situ key generation in extreme environmental conditions. Furthermore, comprehensive security evaluations demonstrated that the proposed reconfigurable PUF exhibits strong resilience against machine learning modeling attacks with prediction accuracies maintained near the ideal random‐guessing level (∼50%) across five classical models (Figure 2d). Furthermore, the sample exhibited exceptional long‐term stability by featuring a data retention of over 10 years and an endurance of up to $1\times 10^{18}$ cycles [56], thereby providing a reliable foundation for high‐frequency security updates. Combined with the 128‐kb capacity and dynamic reconfigurability, our proposal ensures robust scalability for secure IoT deployments.

As a forward‐looking solution, our reconfigurable PUF provides scalable reconfigurability in both the spatial domain (from 1 b to 128 kb) and the temporal domain (on‐demand refresh of CRPs) and thus provides a flexible security solution for dynamic operating environments. The design mitigates temperature‐induced write reconfiguration issues while providing a feasible solution for hardware‐based security, which paves the way for implementation in IoT applications.

## 4 Materials and Methods

### 4.1 Fabrication Processes

The film stack and SOT‐MRAM chip were fabricated in‐house. The optimized W/CoFeB/MgO/CoFeB/SAF system was adopted for the film stack [54]. A top‐pinned structure was adopted for the MTJ, which was prepared above M5V5 as the last level of metal and via in the front‐end‐of‐line (FEOL) substrate. The pillars of the MTJ had a short axis of ∼300 nm and an aspect ratio of ∼2. One basic unit comprised two MTJs for storage and reading. The film stack could tolerate temperatures up to 400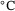, which was typical for solder reflow. The SOT track passed the electro‐migration (EM) and stress migration tests [47]. The chemico‐mechanical polishing process was used to reduce the roughness of the FEOL substrate for deposition of a high‐quality film stack. The etching process was improved by adjusting the plasma conditions and etching angle to alleviate the footing issue. A fine device morphology was achieved, and redeposition of the etch on the sidewall was avoided.

### 4.2 Electrical Measurement

The 128 kb SOT‐MRAM chip supported both digital and analog signal modes. The multiplexer mode enables access to a specific SOT‐MTJ in the analog mode (Figure S2). In this mode, voltages could be applied separately between the bit line and source line, which enables flexible control. The read/write path could also be monitored via current sensing. Under normalized operation conditions, the current flowing through the selected basic unit was calculated and then converted to the resistance of the selected path. The overall array was characterized, and the statistical values were finally obtained. The different write voltages were applied via the analog I/O. The data were read in digital signal mode. Both modes supported variable temperature measurements. All evaluations at the chip level were carried out by using the chip‐probing test platform. Detailed statistics of device‐to‐device variations are provided in Section S2.

## Author Contributions

M. W., C. J., and Z. W. conceived and designed the experiments; C. J., H. L., and Z. Z. conducted the experiment and data acquisition; M. W., W. Z., Z. H., and Y. Z. analyzed the data; All authors discussed the results; M. W. wrote the draft, and all authors contributed to the review and editing; Z. W., H. L., and W. Z. supervised the project.

## Funding

This work was supported by the National Key Research and Development Program of China (Grant No. 2021YFB3601303), the National Natural Science Foundation of China (No. 62171013) and the National Program for Support of Top‐notch Young Professionals.

## Conflicts of Interest

The authors declare no conflicts of interest.

## Supporting information


**Supporting File**: advs74926‐sup‐0001‐SuppMat.pdf.

## Data Availability

The data that support the findings of this study are available from the corresponding author upon reasonable request.
