# Supplementary material for: Temperature‐Resilient Reconfigurable Physical Unclonable Function Driven by Pulse Modulation Using CMOS‐Integrated Spintronic Chips
Source: Adv Sci (Weinh). 2026 Mar 27;13(37):e74926. doi: 10.1002/advs.74926 (PMC13326010; doi:10.1002/advs.74926)
Supplement: Supplementary file 1 — Supporting File: advs74926‐sup‐0001‐SuppMat.pdf. [file ADVS-13-e74926-s001.pdf]

Supplementary Materials:
Temperature-Resilient Reconfigurable Physical Unclonable Function Driven by Pulse Modulation Using CMOS-Integrated Spintronic Chips

This PDF file includes:

Supplementary Text;
Figs. S1 to S22;
Tables S1 to S7.

Contents

S1 Write shmoo of the SOT-MRAM chip 3

S2 Device-to-device process variation 5

S3 Introduction of XOR operation 7

S4 Robustness against machine learning attacks 10

S5 Main metrics of PUF design 12

S6 Illustration of the origin of reconfigurability 17

S7 Reconfigurability in 00-FF-00 polarized writing 22

S8 Temperature independence of SOT-track resistance 24

S9 Reconfigurability under various working temperatures 25

S10 Demonstration of read reliability 26

S11 Issues reflected by slope k 28

S12 Explanation of the dual-Pulse modeling 30

S13 Batch demonstration of the selected β 33

S14 Energy Scaling Analysis 34

S15 Chip architecture optimization perspective 35

S1 Write shmoo of the SOT-MRAM chip

We conducted writing/reading operations under various working temperatures, ranging from -40°C to 125°C, which aligns with the criteria for industrial products. Figure S1 presents the measured write success rate (WSR) of the SOT-MRAM chip in multiplexer testing mode. The WSR means the ratio of the number of successfully switched bits to the total number of bits when applying a specific write voltage and pulse width to the chip. This statistical result is based on all 131,072 units, ultimately yielding statistics for a single pixel.

Write operations of Fig. S1 are implemented via multiplexer mode, with the two MTJs within the unit receiving opposite write voltages. The labeled voltage represents the write voltage for the data bit, while the write voltage for the reference bit is the opposite voltage (reversed polarity). Subsequently, the read operation is carried out in digital mode for statistical analysis of WSR results. Experimental results in Fig. S1 demonstrate the chip's functionality across the temperature range.

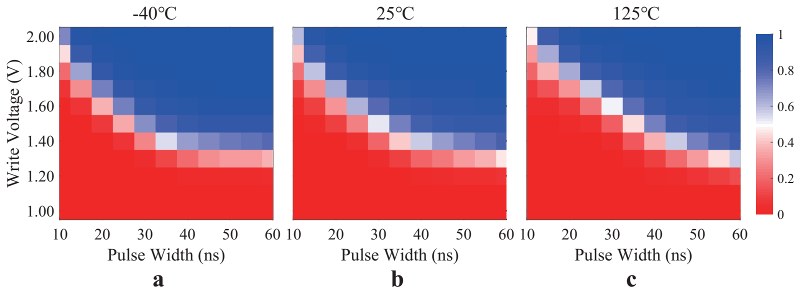

Fig. S1 Write shmoo of SOT-MRAM chip under various pulse widths and write voltages. Working temperature: (**a**) –40°C; (**b**) 25°C; (**c**) 125°C. The color-mapping refers to the write success rate (WSR).

A more detailed explanation of the multiplexer testing mode is as follows, with the schematic of the unit shown in Fig. S2(a). The multiplexer mode enables access to a specific SOT-MTJ in analog mode, where the bit line (BL) and source line (SL) can be voltage-applied, corresponding to the analog I/O of AOUT[1:0] in Figure 1a. Each MTJ is controlled by two transistors, i.e., a read transistor and a write transistor. By controlling the RWL or WWL, the voltage can be applied to either the read path (Fig. S2(b)) or write path (Fig. S2(c)). The read/write path can also be monitored via current sensing. For example, the read path for MTJA is defined as BL1–read transistor–SL1, while the write path is BL1–write transistor–SL1. Factors like global addressing and module paths are assumed negligible, as their resistance values are relatively low.

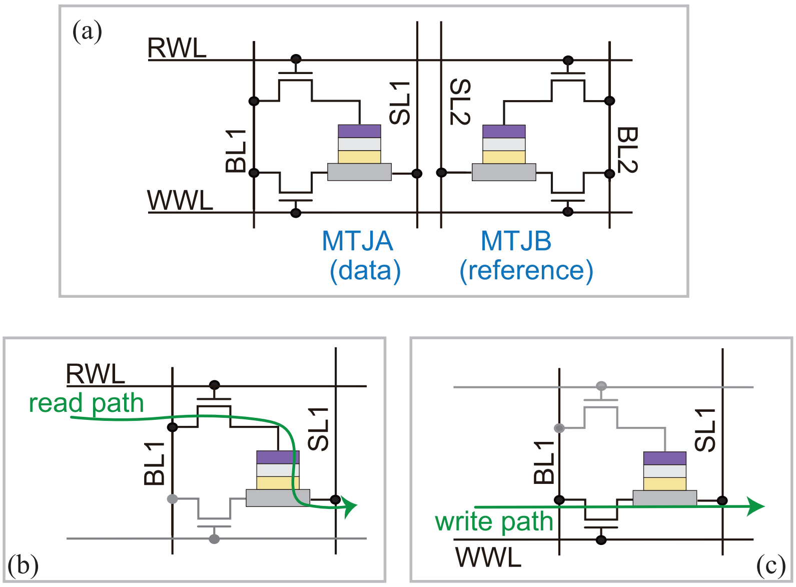

Fig. S2 Illustration of the unit. (**a**) The SOT-MTJ pair and their access transistors in one unit; (**b**) Schematic of read path; (**c**) Schematic of write path.

S2 Device-to-device process variation

Figure S3(a)-(b) refers to the statistics of P-state resistances of all MTJs in the chip, while Fig. S3(c)-(d) presents the statistical results for the AP-state resistance.

Fig. S3(a) displays the resistance distributions of MTJA (data bit) and MTJB (reference bit). Both exhibit near-Gaussian distributions, reflecting inherent device-to-device process variations. Such resistance variations can serve as static entropy sources in PUF implementation. As shown in Fig. S3 (b), when comparing MTJA and MTJB in P states, the resistance difference exhibits tight clustering with a mean (μ) of 97Ω, approaching zero. The average difference in the case of AP states in Fig. S3(d) is 584 Ω, still two orders of magnitude smaller than R_p.The above-mentioned results indicate that comparisons of units within the same state yield a nearly 50% "0/1" distribution. In other words, the process variations of MTJ resistances within SOT-MRAM chips are sufficient to support static PUF implementation.

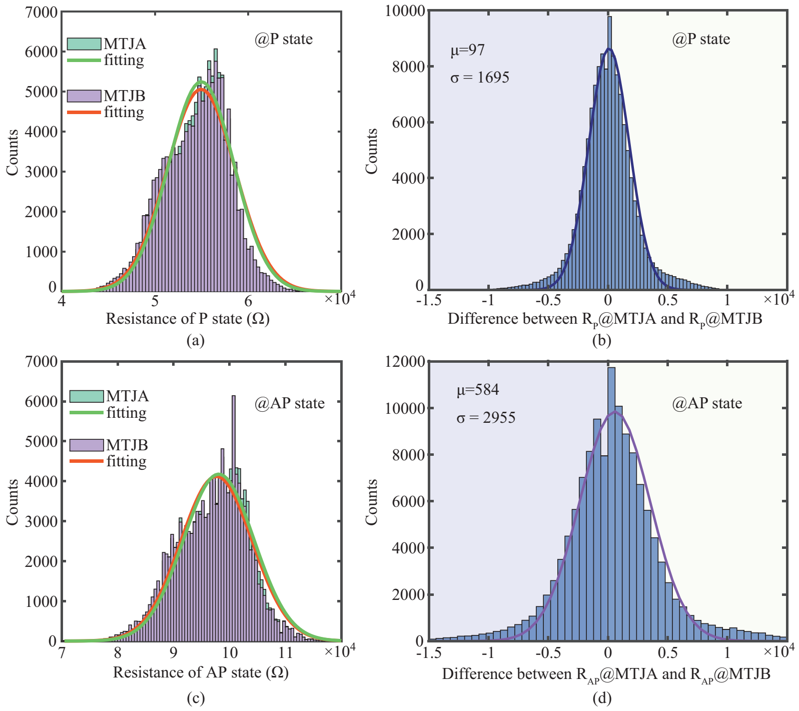

Fig. S3 D2D process variations of MTJ resistances in chips. (**a**) Statistics of P-state resistance for all MTJAs and MTJBs. Lines indicate Gaussian distribution fitting. (**b**) Statistics for differences between $R_P@MTJA$ and $R_P@MTJB$. (**c**)–(**d**) refer to the case of the AP state.

Regarding the coercive field (H_c), this chip-level study primarily focuses on the electrical behaviors, and the D2D variation can be reflected in WSR curves in Figure 2a and shmoo in Figure S1. The device-to-device variation of H_c is demonstrated through testing and analysis of a 1024-bit small-scale array. As shown in Fig. S4 (a),

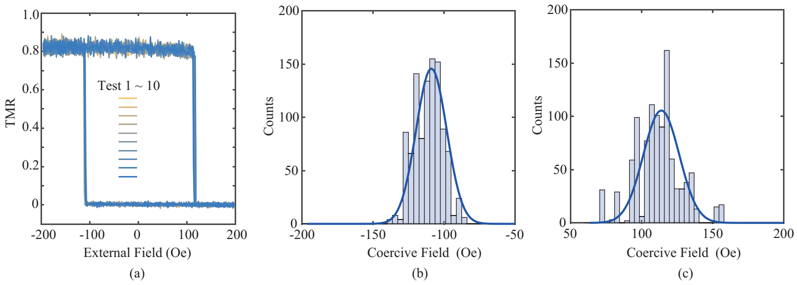

Fig. S4 D2D process variation of coercive field H_c in SOT devices. (**a**) Measurement results of ten R-H curves in a single device. Statistics of H_c for **b** P-state switching and **(b)** AP-state switching, respectively.

each device performs 10 measurements and calculates the average; the measurement deviation of the device itself is relatively small. The H_c of P-state switching and AP-state switching are summarized in Figure S4(b) and (c), respectively. The mean values of the H_c are close to 110 Oe. Statistics resembling a Gaussian distribution reflect device-to-device process variation of H_c.

S3 Introduction of XOR operation

XOR-based post-processing is a widely used technique for improving PUF metrics, such as uniformity, uniqueness, etc. XOR operations with different bit numbers influence uniformity evaluation, where μ and σ are obtained by fitting the histogram. A response is defined as 128 bits, thus the ideal μ and σ are:

$$Ideal\ \mu = 0.5 \tag{S1}$$

$$Ideal\ \sigma = \frac{1}{2\sqrt{128}} \simeq 0.0442 \tag{S2}$$

Figure S5 and S6 refer to uniformity evaluation of PUF responses generated by the single-pulse writing strategy and dual-pulse writing strategy, respectively. It could be observed that:

Firstly, as the bit number in the XOR operation increases, both σ and μ show a trend of improvement. Meanwhile, it should be noted that the modified results also show a slight fluctuation according to the original data.

Secondly, the even/odd dependence. When the number of bits is even, the XOR operation eliminates the counting of parallel and anti-parallel states. For example, $1 \oplus 1 = 0$, $0 \oplus 0 = 0$. Conversely, when the number of bits is odd, this difference is preserved. For instance, $1 \oplus 1 \oplus 1 = 1$, $0 \oplus 0 \oplus 0 = 0$. In wide-temperature-range measurement, the 7-bit XOR operation is finally adopted.

Thirdly, the uniformity evaluation in the case of dual-pulse writing (see Fig. S6) shows better performance compared with that in single-pulse writing (see Fig. S5).

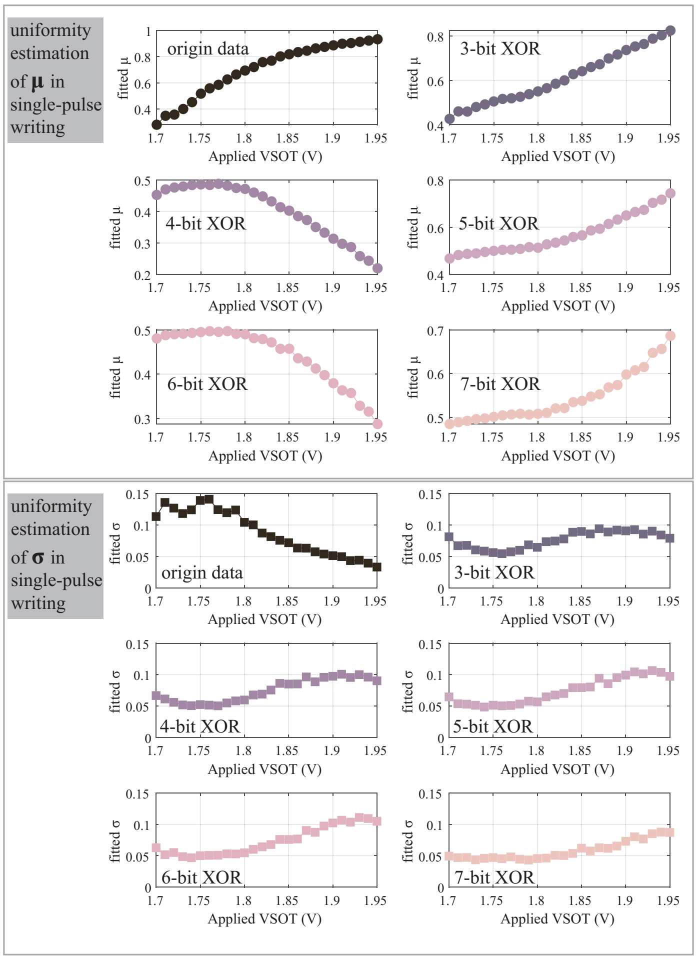

Fig. S5 Fitted μ and σ in uniformity evaluation with various XOR processing. The single-pulse writing strategy is applied.

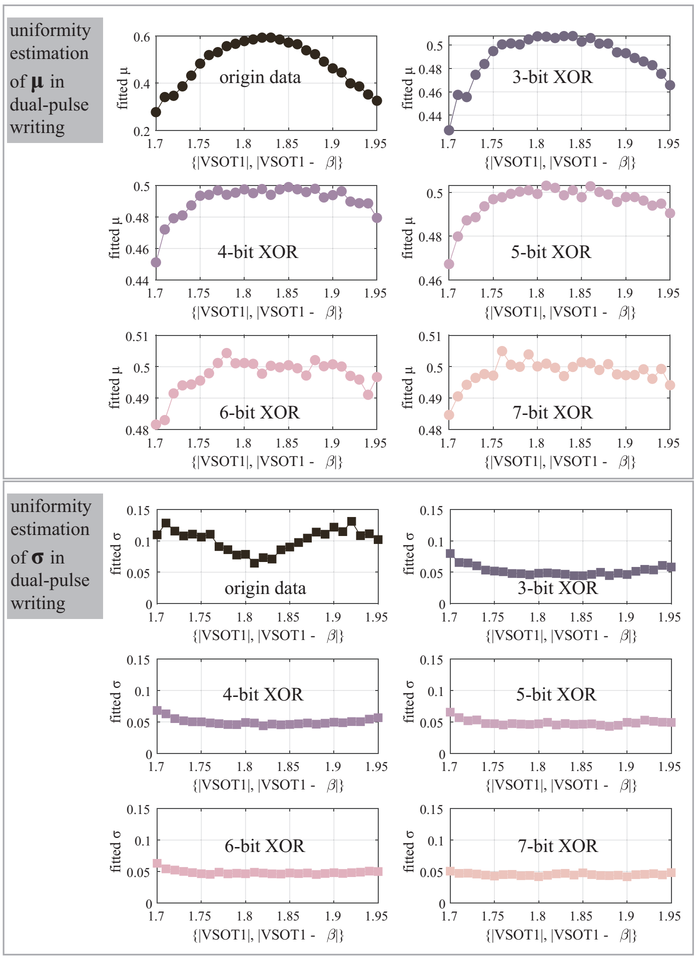

Fig. S6 Fitted μ and σ in uniformity evaluation with various XOR processing. The dual-pulse writing strategy is applied.

S4 Robustness against machine learning attacks

The current architecture and read/write methods possess resistance to side-channel attacks (SCA). The used attack evaluation targets breaking the PUF response mapping. The reasons are as follows:

(i) Secure read operation against SCA: Our chip employs a differential read-out scheme in digital mode, determining data by comparing the resistance values of complementary pairs. For units storing either 0 or 1, externally detectable power consumption or delay variations are nearly identical. Attackers cannot infer the internal analog state of the MTJ from changes in current or power consumption.

(ii) Secure write operation against SCA: In SOT-MRAM, the write current flows through the SOT channel, rather than through the MTJ itself. The channel resistance remains independent of the magnetization state (P or AP-state), rendering the write current curve incapable of revealing stored data. This eliminates the correlation between stored data and write power consumption, significantly reducing side-channel leakage risks.

During normal operation (e.g., Figure S1), both write operations in analog mode and digital read operations are employed; each operation holds security against channel attacks. Therefore, our evaluation focuses on whether machine learning algorithms can learn the mapping relationship between challenges and responses, thereby validating the security of the proposed PUF.

Five ML attacks are investigated, including support vector machine (SVM), random forest, artificial neural network (ANN), logistic regression (LR), and Covariance Matrix Adaptation Evolution Strategy (CMA-ES). In terms of machine learning attacks, the PUF data is randomly divided into a training set and a test set, and the ratio of the two is 7:3. The training set is used for training and iteration, and the test set is used to determine the accuracy of machine learning predictions. The following are the details of those attack methods.

1. Logic Regression (LR) Attack. The LR attack uses a sigmoid function to discriminate the output and improve the accuracy of the prediction. Ridge regression is used to prevent overfitting, and the LBFGS algorithm optimizes the parameters used in the model.

2. Support Vector Machine (SVM) Attack. SVM achieves partitioning of data by finding an optimal hyperplane. The kernel function is set to Radial Basis Function (RBF). Regularization parameter and Kernel coefficient are determined through grid search with 3-fold cross-validation.

3. Covariance Matrix Adaptation Evolution Strategy (CAM-ES). During the iterative process, suitable parameters and models will “naturally evolve”. The core approach is to evolve the model towards an optimal solution through parameter tuning. The number of XOR operations used in each stage is 1, which is the typical operation in the attack against PUF. Fitness function refers to the method introduced in Ref. [1].

4. Artificial Neural Network (ANN) Attack. ANN mimics the connections between biological neurons. Here we employ the Multi-Layer Perceptron (MLP) classifier, consisting of the input layer, the hidden layer with 100 neurons, and the output layer. This method uses L2 regularization, ReLu activation function, and LBFGS as the solver.

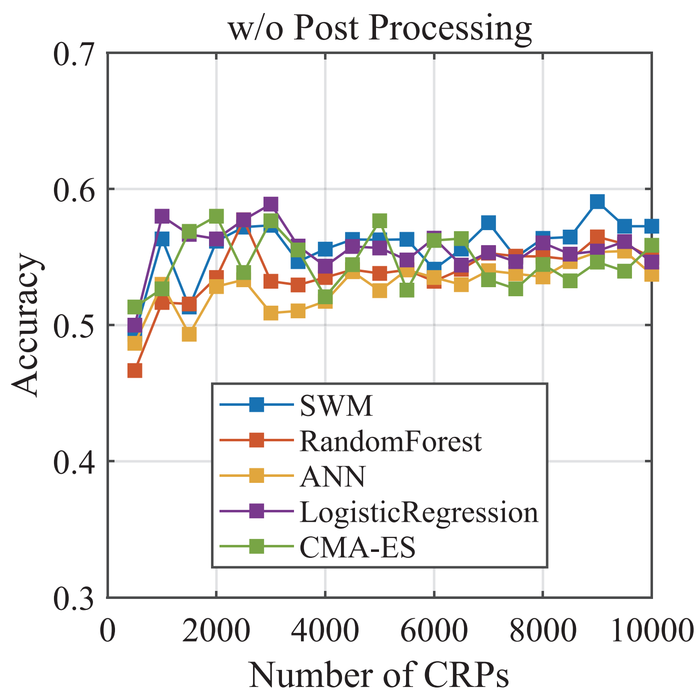

Fig. S7 Machine learning results for the training data without XOR operation.

5. Random Forest (RF) Attack. Based on the Bagging algorithm, RF frequently creates multiple new classifiers, forming decision trees. The measure of split quality uses information gain (entropy). The out-of-bag (OOB) samples are allowed for assessment of model performance.

The ML attack results for data without XOR post-processing are evaluated as 0.5529 (see Fig. S7).

S5 Main metrics of PUF design

The following presents the evaluation of the uniformity of the SOT-MRAM rPUF, with a PUF response defined as 128 bits.

Figure S8 refers to the uniformity evaluation of 00-FF polarized single-pulse and 00-FF-00 polarized dual-pulse reconfiguration.

Figure S9 refers to the uniformity evaluation after FF-00 polarized single-pulse and FF-00-FF polarized dual-pulse reconfiguration.

It is observed that the dual-pulse writing effectively broadened the operation window width. The uniformity of PUF responses is further enhanced via the XOR operation. As a result, more intensive distributions and reduced σ in Gauss fitting are achieved by the dual-pulse method and XOR operation in Fig. S8 and S9. Here, the 3-bit XOR operation is used to show a clear trend in $sigma$ values. In the use of 7-bit XOR, the uniformity after dual-pulse writing is estimated as 0.5001 as fitted μ, with 0.042 as fitted σ.

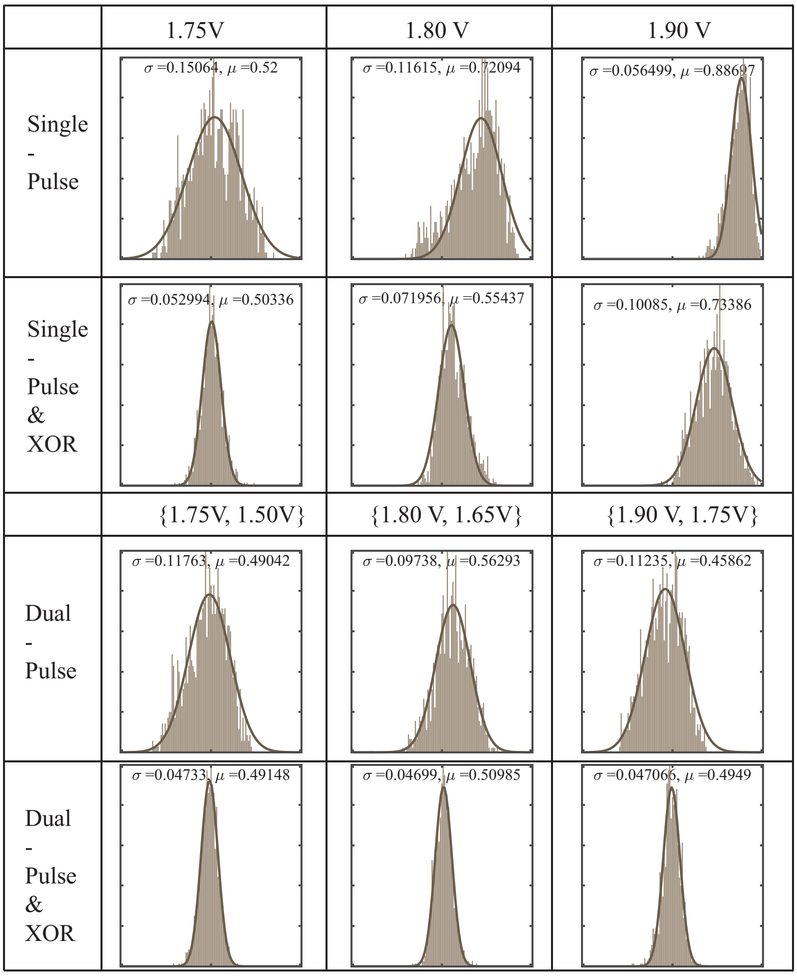

Fig. S8 Uniformity of SOT-MRAM rPUF in 00-FF and 00-FF-00 polarized reconfiguration.

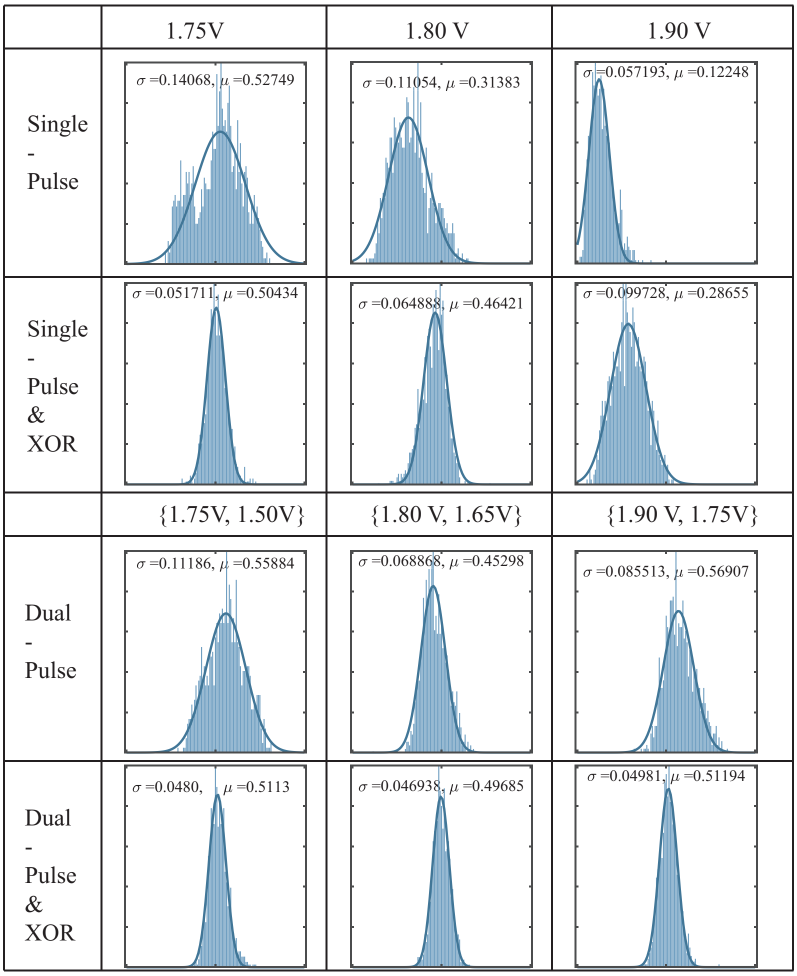

Fig. S9 Uniformity of SOT-MRAM rPUF in FF-00 and FF-00-FF polarized reconfiguration.

Fig. S10 shows the result of the Auto-Correction Function (ACF) test, where the majority of data points are located within the confidence range interval. The ACF test result confirms the unpredictability and randomness of reconfiguration in our proposed rPUF design.

Table S1 lists the NIST SP800-22 test result, confirming the randomness of reconfigured CRPs by the proposed rPUF design.

The intra-HD and inter-die HD are shown in Fig. S11. Fig. S11a shows 3 cases of intra Hamming distance (HD): direct readout, time-majority-vote (TMV), and self-write-back (SWB). Fig. S11b exhibits good uniqueness, evaluated by the calculation of the inter-die HD. The Inter/Intra-HD ratio approximates ∞ with the aid of the SWB technique, estimated as $\sim$1500 $\times$.

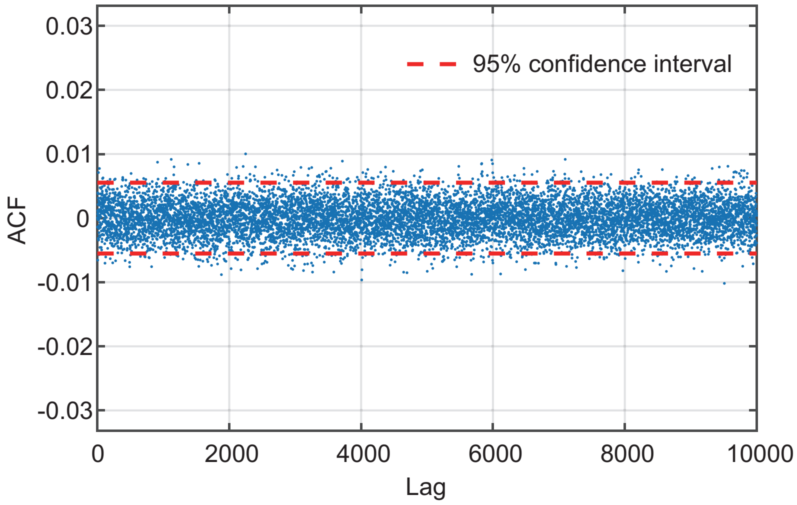

Fig. S10 Auto-correlation Function (ACF) testing of the measured PUF responses.

Table S1 NIST SP800-22 test result

Test	p-Value	Proportion	Pass?
Frequency	0.122325	10/10	YES
BlockFrequency	0.911413	10/10	YES
CumulativeSums-1	0.911413	9/10	YES
CumulativeSums-2	0.911413	9/10	YES
Runs	0.534146	9/10	YES
LongestRun	0.122325	10/10	YES
Rank	0.350485	10/10	YES
FFT	0.534146	10/10	YES
NonOverlappingTemplate	PASS	PASS	YES
OverlappingTemplate	0.350485	9/10	YES
ApproximateEntropy	0.017912	10/10	YES
Serial-1	0.534146	10/10	YES
Serial-2	0.534146	10/10	YES
LinearComplexity	0.350485	9/10	YES

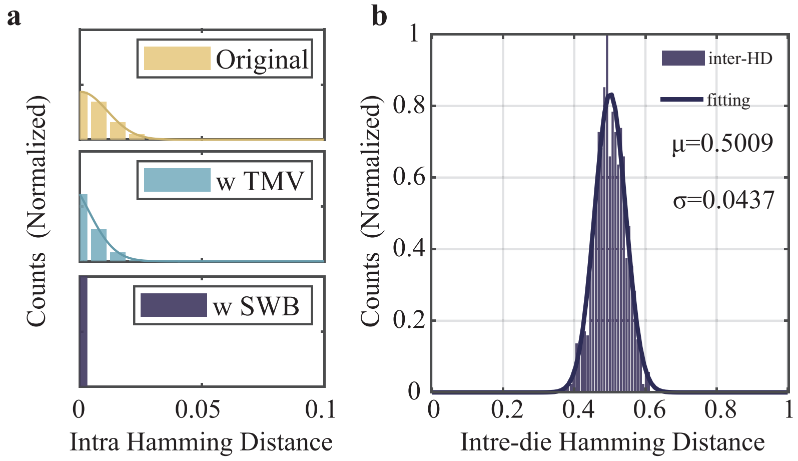

Fig. S11 (**a**) Intra HD: direct readout, time-majority-vote (TMV), and self-write-back (SWB). (**b**) Inter-die HD distribution with the mean value near-ideal 50%.

S6 Illustration of the origin of reconfigurability

S6.1 Comprehension of C2C and D2D variability

The spatial distribution of PUF data is the union of all units with certain time-domain data. In the performance of reconfiguration, device-to-device (D2D) variation shows a mapping relationship with the statistical behavior of cycle-to-cycle (C2C) variation.

In Fig. S12, the first column displays two statistical maps of switching probabilities, with polarity of 00-FF-00 and FF-00-FF, respectively. The switching probabilities for each unit are derived from 50 reconfigurations. At one of these reconfigurations, the chip presents a binary PUF data distribution. The second column counts the uniformity of the PUF response after a certain reconfiguration. The first two rows of the second column are from the raw data based on the first statistical map. The third row of the second column corresponds to the second statistical map. The third column indicates the uniqueness between reconfigurations guided by the connected arrow.

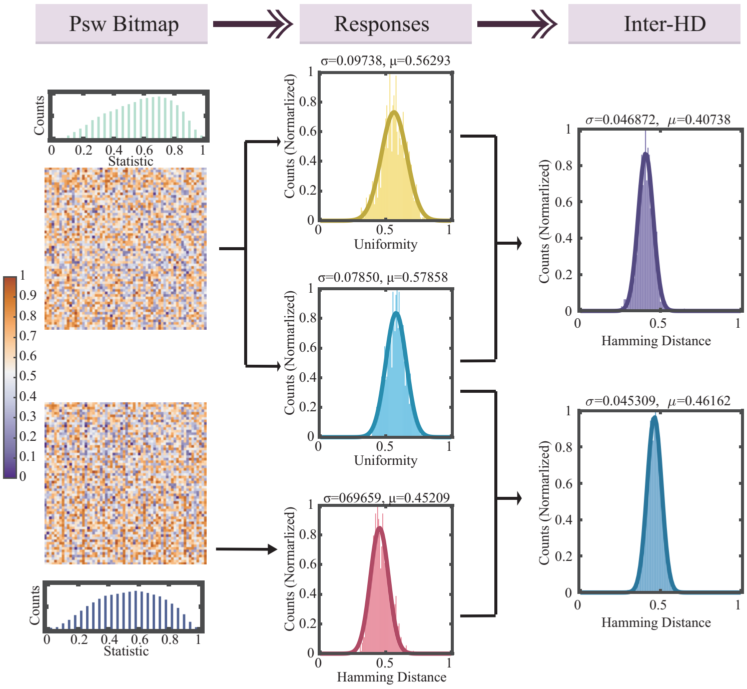

Fig. S12 Relationship between Psw statistic map, uniformity of responses, and Inter-Reconfig. HD.

The reconfiguration originating from different Psw map results shows better Inter-Reconfig. HD. The Inter-Reconfig. HD. by the same-polarized writing (i.e., from the same Psw map) is not exactly ideal; meanwhile, this result provides a prerequisite for XOR operations. Under the aid of the XOR operation, reconfigurations originating from different/same Psw maps are both available for the formation of separate PUFs.

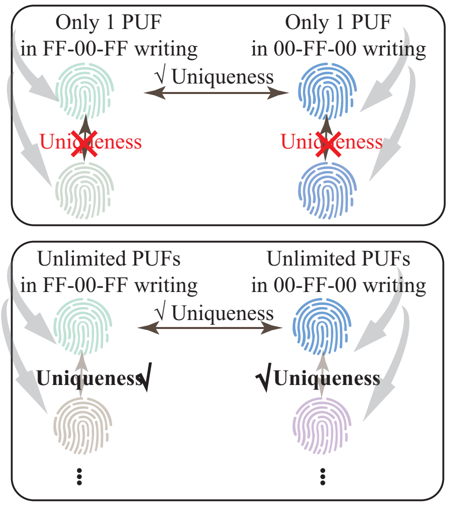

Fig. S13 Unlimited PUF reconfiguration in aid of XOR operation.

As shown in Fig. S13, the SOT-MRAM rPUF was successfully validated for unlimited reconfiguration.

The above descriptions and illustrations help the understanding of Fig. 3 in the main manuscript.

S6.2 Quantitative evaluation of reconfigurability

Table S2 refers to the evaluation of reconfigurability: two cases of PUF responses generated by the same polarity but without XOR.

Table S3 refers to the evaluation of reconfigurability: two cases of PUF responses generated by the same polarity and with XOR.

Table S4 refers to the evaluation of reconfigurability: two cases of PUF responses generated by the opposite polarity but without XOR.

Table S5 refers to the evaluation of reconfigurability: two cases of PUF responses generated by the opposite polarity and with XOR.

Table S2 Reconfigurations with the same polarity but without XOR

Method	Statistic of Psw @case1	Statistic of Psw @case2	Inter-reconfig. HD	Distance from 0.5
Single-Pulse	00-FF<br>$\mu = 0.7459$ σ=0.2048	00-FF<br>$\mu = 0.7459$ σ=0.2048	0.3340	0.166
Dual-Pulse	00-FF-00<br>$\mu = 0.5734$ σ=0.2057	00-FF-00<br>$\mu = 0.5734$ σ=0.2057	0.4076	0.0924
Single-Pulse	FF-00<br>$\mu = 0.7183$ σ=0.2154	FF-00<br>$\mu = 0.7183$ σ=0.2154	0.3556	0.1444
Dual-Pulse	FF-00-FF<br>$\mu = 0.5356$ σ=0.2120	FF-00-FF<br>$\mu = 0.5356$ σ=0.2120	0.4152	0.0848

Table S3 Reconfigurations with the same polarity and with XOR

Method	Statistic of Psw @case1		Statistic of Psw @case2		Inter-reconfig. HD	Distance from 0.5
Single-Pulse + XOR	00-FF		FF-00		0.4709	0.0291
	$\mu = 0.5987$	σ=0.1405	$\mu = 0.5987$	σ=0.1405		
Dual-Pulse + XOR	00-FF-00		FF-00-FF		0.4972	0.0028
	$\mu = 0.5094$	σ=0.0818	$\mu = 0.5094$	σ=0.0818		
Single-Pulse + XOR	FF-00		FF-00		0.4813	0.0187
	$\mu = 0.5772$	σ=0.1319	$\mu = 0.5772$	σ=0.1319		
Dual-Pulse + XOR	FF-00-FF		FF-00-FF		0.4957	0.0043
	$\mu = 0.5040$	σ=0.0819	$\mu = 0.5040$	σ=0.0819		

Table S4 Reconfigurations with the opposite polarity but without XOR

Method	Statistic of Psw @case1	Statistic of Psw @case2	Inter-reconfig. HD	Distance from 0.5
Single-Pulse	00-FF<br>μ = 0.7459 σ=0.2048	00-FF<br>μ = 0.7183 σ=0.2154	0.5657	0.0657
Dual-Pulse	00-FF-00<br>μ = 0.5734 σ=0.2057	00-FF-00<br>μ = 0.5346 σ=0.2120	0.4619	0.0381

Table S5 Reconfigurations with the opposite polarity and with XOR

Method	Statistic of Psw @case1	Statistic of Psw @case2	Inter-reconfig. HD	Distance from 0.5
Single-Pulse + XOR	00-FF<br>μ = 0.5987 σ=0.1405	FF-00<br>μ = 0.5772 σ=0.1319	0.5068	0.0068
Dual-Pulse + XOR	00-FF-00<br>μ = 0.5094 σ=0.0818	FF-00-FF<br>μ = 0.5040 σ=0.0819	0.4990	0.0001

S7 Reconfigurability in 00-FF-00 polarized writing

Reconfigurability is defined as Inter-Reconfig. HD. The 00-FF-00 polarized reconfiguration is shown in Fig. S14, exhibiting a similar tendency to the FF-00-FF polarized reconfiguration. The combination of 00-FF-00 dual-pulse writing and XOR post-processing leads to unlimited reconfiguration counts and a large operation window.

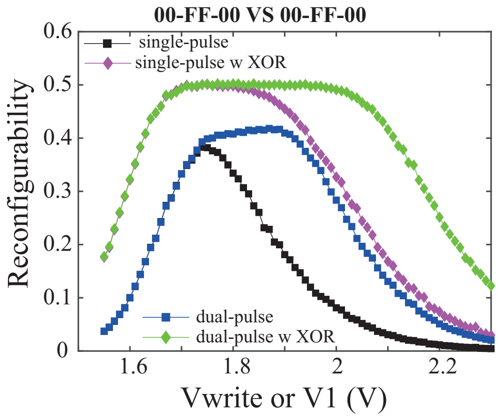

Fig. S14 Reconfigurability (Inter-Reconfig. HD) as a function of write voltage amplitudes.

Conducting over 50 counts of 00-FF-00 polarized reconfiguration, good reconfigurability is demonstrated in Fig. S15.

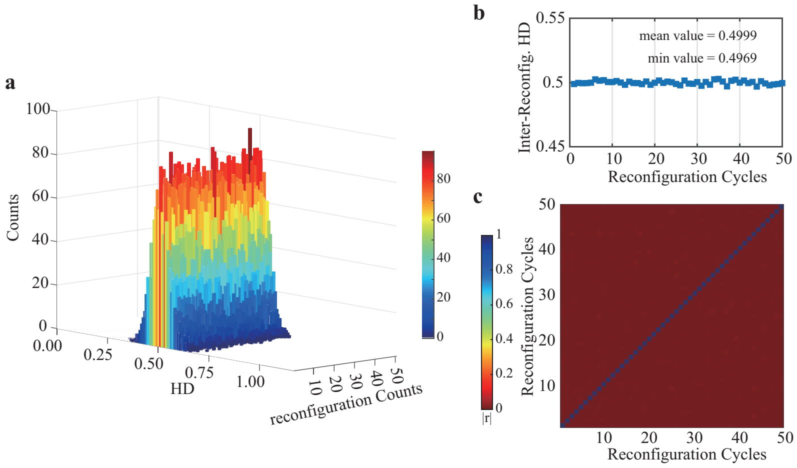

Fig. S15 Evaluation of reconfigurability considering 00-FF-00 polarized reconfigurations. (**a**) Distribution of the normalized Inter-reconfig. HDs over 50 reconfigurations. (**b**) Statistics of the mean value of Inter-reconfig. HD. (**c**) Correlation matrix concerning the reconfiguration results.

S8 Temperature independence of SOT-track resistance

The material of the SOT track is mainly annealed β-phase tungsten (β-W). We tested the resistance of the SOT track in single SOT-MTJs, which are fabricated on the same platform but without CMOS. The resistance values of the SOT track at various temperatures are listed in Table S6. The results indicate that the SOT track resistance of all samples exhibits negligible dependence on temperature. The minor fluctuations observed could be attributed to measurement error. Consequently, it is demonstrated that the resistivity of annealed β-W is essentially independent of temperature, consistent with the previous report[2].

Table S6 Resistance of SOT track measured under various temperatures

	**-40°C**	**0°C**	**25°C**	**75°C**	**125°C**
SOT-MTJ #1	676	681	678	678	673
SOT-MTJ #2	741	741	743	740	742
SOT-MTJ #3	672	674	678	674	670
SOT-MTJ #4	679	680	685	681	680
SOT-MTJ #5	706	710	712	707	704
SOT-MTJ #6	741	744	744	738	741
SOT-MTJ #7	829	832	831	825	826
SOT-MTJ #8	786	786	790	783	783
SOT-MTJ #9	719	722	722	721	718
SOT-MTJ #10	698	700	700	700	699
SOT-MTJ #11	672	675	674	670	671
SOT-MTJ #12	681	684	685	682	688
SOT-MTJ #13	706	709	711	708	717
SOT-MTJ #14	750	753	753	750	751

S9 Reconfigurability under various working temperatures

Figure S16a shows the Inter-Reconfig. HD between the reconfiguration under 25°C and that under 125°C. Figure S16b shows the Inter-Reconfig. HD between the reconfiguration under 25°C and that under -40°C. Reconfigurability under various working temperatures proves the feasibility of unified reconfiguration settings. There is no need for sensor feedback or waiting for the environment to drop to room temperature. Therefore, the dual-pulse strategy increases the feasibility of operation and releases the need for a temperature feedback circuit module, showing excellent potential in hardware security.

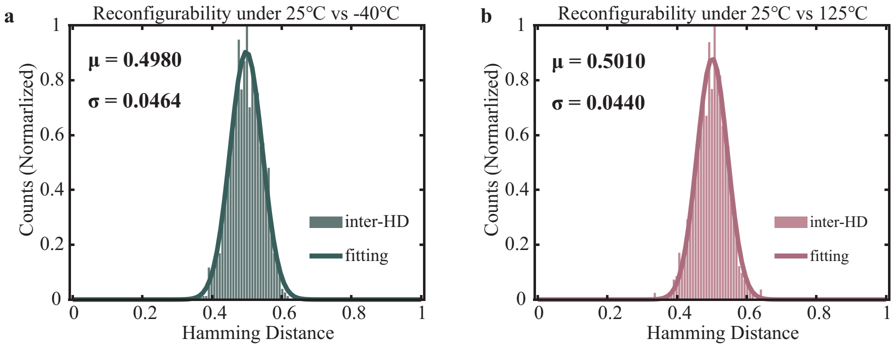

Fig. S16 Evaluation of Inter-Reconfig. HD: (**a**) between the reconfiguration under 25°C and that under -40°C, (**b**) between the reconfiguration under 25°C and that under 125°C.

S10 Demonstration of read reliability

Bit Error Rate (BER) indicates the reliability of the data readout. The nominal conditions are defined as room temperature (25°C) and 1.8 V supply voltage, which refers to the supply voltage in the digital module that is mainly used for addressing, controlling the read transistor, etc.

Figure S17 and S18 present the BER result with and without SWB, respectively, and all data are based on the average of 15 times of readout. With the help of SWB, the BER under the nominal condition is estimated to be 3.29×10^{-5}, ∼474 × improved reliability compared to the results w/o SWB. In addition, PUF with SWB also shows good read reliability in the cases of varying temperature and supply voltage.

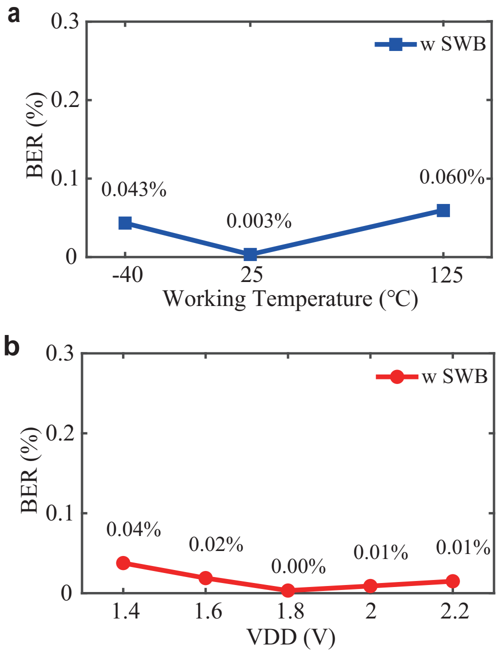

Fig. S17 Evaluation of reliability w SWB operation. BER of the SOT-MRAM rPUF with (**a**) the temperature ranging from -40°C to 125°C, and (**b**) the supply VDD voltage ranging from 1.4 V to 2.2 V.

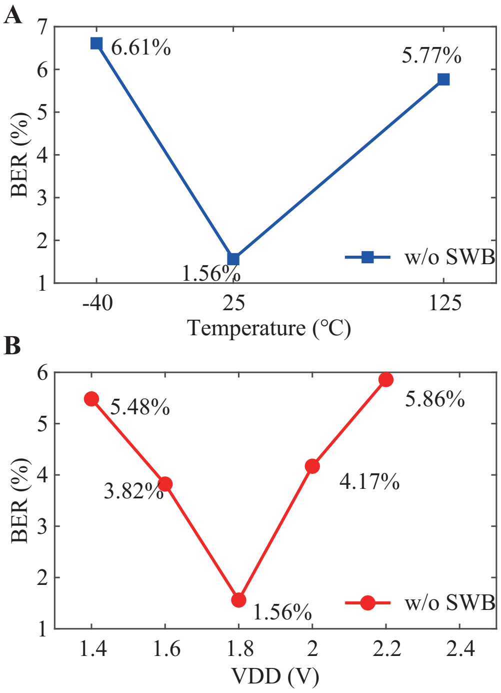

Fig. S18 Evaluation of reliability w/o SWB operation. BER of the SOT-MRAM rPUF with (**a**) the temperature ranging from -40°C to 125°C, and (**b**) the supply VDD voltage ranging from 1.4 V to 2.2 V.

S11 Issues reflected by slope k

The slope k reflects the discrete degree of device performance; meanwhile, the slope k is the essential parameter to solve β.

We perform numerical modeling, with preliminary assumptions of introducing the process variation of the SOT track width. The numerical modeling follows the steps below:

(i) Setting baseline case and calculating the initial critical current J_{c0}.

(ii) Introducing bottom electrode width variation. Generate the SOT track width for each SOT-MTJ through a random distribution by introducing the coefficient of variation (CV), which is expressed as σ/μ.

(iii) Device-level switching probability curves. For each SOT-MTJ, the switching probability curve as a function of current Psw(V) differs in various SOT track widths.

(iv) Signal-driven switching probability and data generation. Based on the input write signals (e.g., specific current/voltage), query the corresponding switching probability, and generate the storage bits by combining with the uniform distribution.

(v) Hamming weight Statistics. Iterate through all SOT-MTJs and count the percentage of successful switching.

(vi) Multi-signal scanning and curve generation. Vary the write signal parameters (e.g., voltage step size) and repeat steps iv-v to generate the curve of Hamming Weight with the signal.

(vii) Slope k calculation. At Hamming weight = 50%, a linear fit to the curve was performed and the slope k was extracted.

The parameter k is a function of variation and the MTJ magnetic parameters. When the preparation is totally ideal and there are no process deviations, all SOT-MTJs exhibit consistent properties. At this point, the HW curve should coincide with the Psw curve of a single SOT-MTJ, as shown in Fig. S19a. This situation is not possible in practice. When process bias exists, the HW curve aggregates the situation of all SOT-MTJs in the array. When the process deviation is larger, the difference between SOT-MTJs becomes larger and the HW curve rises more slowly, as shown in Fig. S19b-c. For the slope at 50% is extracted and fitted, as shown in Fig. S19d.

The model indicates that k reflects the process variation. It should be noted that Fig. S19 only explains the influence of process variation, where the extracted values are not the experimental results.

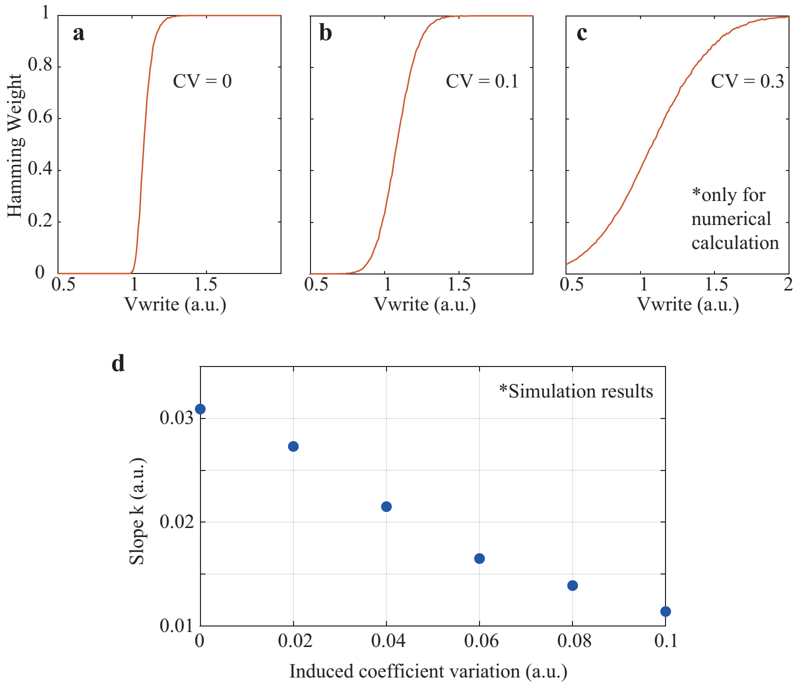

Fig. S19 Numerical prediction of slope k. Hamming weight as a function of applied voltage in the case of (**a**) no process variation, (**b**) with coefficient variation of 0.1, (**c**) coefficient variation of 0.3. The case of (A) equals to the switching probability of the single SOT-MTJ. (**d**) The evaluated k as a function of process variation. The induced coefficient variations are set only for numerical calculation.

S12 Explanation of the dual-Pulse modeling

S12.1 Independent events in the case of the 00-FF and FF-00 pulses

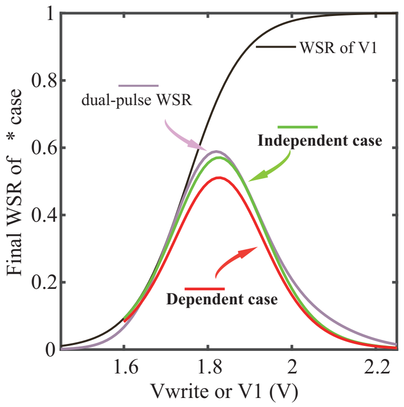

Fig. S20 Verification of dual-pulse modeling. The experimental results align well with the simulated results in dual-pulse reconfiguration. Independent case means Eq. S3. Dependence case refers to Eq. S4.

Figure S20 confirms that the bit switching behaviors in the first and second pulses are independent events. From the unit perspective: assuming that the MTJ holds the switching probability P_1 (P_2) of 00-FF (FF-00) polarized writing when applying only one pulse, the final time-domain distribution probability is expressed as $P_1 - P_1 \times P_2$ when applying 00-FF-00 polarized dual-pulse writing.

From the array perspective, the WSR shows the array-level statistic results in the spatial-domain distribution, reflecting the whole behavior of all single units. Assuming that the array holds the $WSR1$ ($WSR2$) of 00-FF (FF-00) polarized single-pulse writing, the final WSR (noted as F) after applying dual pulses is given by:

$$F = WSR1 - WSR1 \times WSR2 \tag{S3}$$

The modeling in Eq. S3 requires two premises: 1. unit switching processes are independent events; 2. The distribution of switching probability holds randomness.

The following is the case of the dependent event. If $WSR1$ and $WSR2$ hold a dependent relationship in domain distribution, the final WSR (F) would be far smaller than the predicted value. For example, if the units switched by the second pulse are a subset of those affected by the first pulse, the relationship can be expressed as follows:

$$F = WSR1 - WSR2 \tag{S4}$$

As shown in Fig. S20, results of Eq. S3 show a better agreement compared with that of Eq. S4. And the mean absolute error for the model of Eq. S3 and experimental

is about 0.02, which confirms again the strong independent event according to the first and the second pulse writing results.

S12.2 Calculation of β in simplified model

As the verification of the independent case in Sec. S12.1, the final WSR F after two write operations has an algebraic relationship with $WSR1$ and $WSR2$, given by

$$F(V1, V2) = WSR1(V1) - WSR1(V1) \cdot WSR2(V2) \tag{S5}$$

In this subsection, we first makes a simplification of the asymmetry of the two pulses. **Assumption (a)**: Pulses of different polarities have approximate WSRs.

$$WSR1(V) = WSR2(V) \tag{S6}$$

In this case,

$$F(V1, V2) = WSR1(V1) - WSR1(V1) \cdot WSR1(V2) \tag{S7}$$

Since the sigmoid-like Psw function grows sharply around 50%, a local fit can be done with a tangent line to form a unitary function. **Assumption (b)**: WSR1 could be simplified to a tangent line, which is only valid around the critical VSOT.

$$WSR1(V) = k \times V + b \tag{S8}$$

Because of $|V_2| = |V_1| - \beta$, F can be expressed as

$$\begin{aligned} F(V1, V2) &= WSR1(V1) - WSR1(V1) \cdot WSR1(V1 - \beta) &\quad \text{(S9)} \\ &= WSR1(V1) - WSR1(V1) \cdot (WSR1(V1) - k\beta) &\quad \text{(S10)} \end{aligned}$$

then,

$$\begin{aligned} F(V1, V2) &= (1 + k\beta) \cdot WSR1(V1) - WSR1(V1)^2 &\quad \text{(S11)} \\ &= -k^2V1^2 + (k + k^2\beta - 2kb) \cdot V1 + (b + k\beta b - b^2) &\quad \text{(S12)} \end{aligned}$$

To solve for the value of β, the extreme value could be resolved as:

$$F_{extreme} = \frac{1}{4}(1 + k\beta)^2 \tag{S13}$$

Here we define the available value of β are those values making $F_{extreme}$ within the target window, for a continuous and large operating window of available voltage, which also corresponds to the region in trend (ii) in the main manuscript. A target window of (0.4, 0.6] is used, which is the result without the XOR operation, and the post-processing would adjust the results within this window to a more ideal range. By solving $0.4 < F_{extreme} \leq 0.6$,

$$\beta \in [-0.6829, -0.6068) \cup (0.0710, 0.1471] \tag{S14}$$

Since Assumption (b) is only valid around the critical VSOT, the solution of [-0.6829, -0.6068) is discarded, and (0.0710, 0.1471] is valid.

Thus, the available β is (0.0710, 0.1471].

$$\beta \in (0.0710, 0.1471] \tag{S15}$$

The optimized case means that the extreme value is equal to the boundary of the target window.

$$F_{extreme} = Upperbound \tag{S16}$$

Therefore,

$$\boxed{Optimal\ \beta = 0.147\ V} \tag{S17}$$

S12.3 Extended modeling

If we also consider asymmetries, offsets, etc., in our chip, we can modify the simplified model of the previous subsection, with different slopes and intercepts,

$$WSR1(V) = k_1 \times V + b_1 \tag{S18}$$

$$WSR2(V) = k_2 \times V + b_2 \tag{S19}$$

F is expressed as $WSR1(|V_1|) - WSR1(|V_1|) \cdot WSR2(|V_1| - \beta)$. The rest of the computational procedure is similar to the previous subsection.

S13 Batch demonstration of the selected β

β is demonstrated to be a stable parameter for SOT-MRAM chips within the same batch. Table S7 lists wide-temperature-range measured results of 10 chips, all of which show the temperature-resilient reconfiguration. This finding facilitates the chip design and reconfiguration operation.

Table S7 Verification of reconfigurability by applying unified operations in the same batch of SOT-MRAM chips

No. of samples	HW @ -40°C	HW @ 125°C	Reconfigurability
#1	0.50014	0.50041	0.49607
#2	0.49709	0.50224	0.50137
#3	0.49647	0.50275	0.49995
#4	0.49935	0.50413	0.49832
#5	0.49803	0.49905	0.50198
#6	0.49876	0.50076	0.49973
#7	0.49874	0.49651	0.49992
#8	0.49934	0.49882	0.49988
#9	0.49886	0.49990	0.49933
#10	0.50423	0.49822	0.49809

S14 Energy Scaling Analysis

The read operation currently consumes ∼10 pJ per bit, and the write process consumes ∼65 pJ per data bit (with CMOS circuits contributing 42 pJ and MTJ for 23 pJ). The energy consumption is not competitive, primarily constrained by the 180 nm node, large MTJ dimensions (CD ∼300 nm), and 20 ns pulse width. It is foreseeable that significant reductions in energy consumption can be achieved through process scaling and device optimization.

Assuming energy scales with the square of the technology node ($E \propto (Tech)^2$) [3], the read energy consumption is projected to decrease to approximately 0.24 pJ per bit at the 28-nm node. The write energy consumption of the CMOS part would be reduced to ∼ 1.02 pJ. Regarding the scaling of a single SOT-MTJ, the latest production line report data is shown in Figure S21. For two pulses in dual-pulse reconfiguration, the energy consumption of the SOT-MTJ is estimated to be ∼1.57 pJ as the optimal value. Thus, the energy consumption of the write operation is projected to decrease to ∼2.59 pJ.

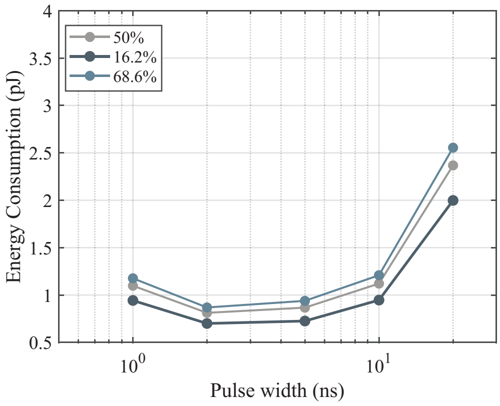

Fig. S21 Energy consumption per SOT-MTJ under various pulse widths. Each line represents the power consumption at the expected switching probability.

S15 Chip architecture optimization perspective

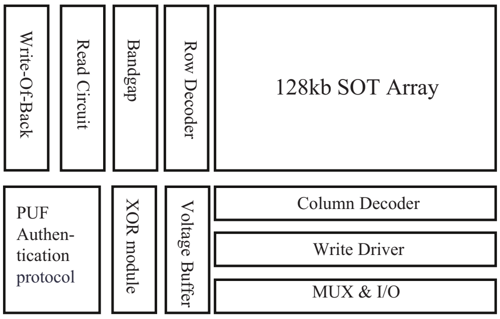

Fig. S22 Chip structure as a perspective.

As a prospect, our proposal is feasible and holds potential to design a multi-mode chip, with a structure shown in Fig. S22. Overall, the integrated chip contains the PUF authentication protocol, a post-processing module, the bandgap circuit, the reference buffer circuit, the read/write circuit, the row/column decoder, I/O, etc. The bandgap circuit can produce a stable reference voltage that does not vary with temperature. The reference buffer circuit outputs a stabilized reference voltage to the write circuit.

Specifically, the amplitude of $V1$ and β can be obtained from the built-in memory of the PUF or deduced from empirical parameters. After that, the reference voltage buffer circuit processes the amplitude of the first pulse and the amplitude of the second pulse based on the reference voltage and outputs two write voltages. Finally, reconfiguration of the PUF is realized after inputting two write voltages to the write driver.

References

[1] Xu, C. et al. Modeling-attack-resistant strong PUF exploiting stagewise obfuscated interconnections with improved reliability. IEEE Internet of Things Journal 10, 16300–16315 (2023).

[2] Hao, Q., Chen, W. & Xiao, G. Beta (β) tungsten thin films: Structure, electron transport, and giant spin Hall effect. Applied Physics Letters 106 (2015).

[3] Xie, S. et al. EDRAM-CIM: Reconfigurable charge domain compute-in-memory design with embedded dynamic random access memory array realizing adaptive data converters. IEEE Journal of Solid-State Circuits 59, 1950–1961 (2023).
